# Supplementary material for: An hepatitis B and D virus infection model using human pluripotent stem cell-derived hepatocytes
Source: EMBO Rep. 2024 Sep 4;25(10):17. doi: 10.1038/s44319-024-00236-0 (PMC11466959; doi:10.1038/s44319-024-00236-0)
Supplement: Supplementary file 1 — Appendix [file 44319_2024_236_MOESM1_ESM.pdf]

Appendix for

An Hepatitis B and D Virus Infection Model Using Human Pluripotent Stem Cell-Derived Hepatocytes

Huanting Chi[1,2,†], Bingqian Qu[1,3,†,§], Angga Prawira[3], Talisa Richardt[3], Lars Maurer[1,4], Jungen Hu[1], Rebecca M. Fu[1], Florian A. Lempp[3,||], Zhenfeng Zhang[3,¶], Dirk Grimm[2,4,5], Xianfang Wu[6], Stephan Urban[2,3,*,‡], and Viet Loan Dao Thi[1,2,*,‡]

[1]Schaller Research Group, Department of Infectious Diseases, Virology, Heidelberg University, Medical Faculty Heidelberg, Heidelberg, Germany; [2]German Centre for Infection Research (DZIF), Partner Site Heidelberg, Heidelberg, Germany; [3]Molecular Virology, Department of Infectious Diseases, Heidelberg University, Medical Faculty Heidelberg, Heidelberg, Germany; [4]Department of Infectious Diseases, Virology, Section Viral Vector Technologies, University Hospital Heidelberg, Cluster of Excellence CellNetworks, BioQuant, Center for Integrative Infectious Diseases Research (CIID), Heidelberg, Germany; [5]German Center for Cardiovascular Research (DZHK), Partner Site Heidelberg, Heidelberg, Germany; [6]Infection Biology Program and Department of Cancer Biology, Lerner Research Institute, Cleveland Clinic Foundation, Cleveland, OH, USA.

Current address:
[§]Division of Veterinary Medicine, Paul-Ehrlich-Institut, Langen, Germany
[||]Humabs Biomed SA, A Subsidiary of Vir Biotechnology, Bellinzona, Switzerland
[¶]School of Public Health and Emergency Management, School of Medicine, Southern University of Science and Technology, Shenzhen, China

[†]equal contribution
[‡]equal contribution
*Corresponding authors: Stephan.Urban@med.uni-heidelberg.de and VietLoan.DaoThi@med.uni-heidelberg.de

Table of Contents:

1. Appendix Table S1 ………. Pages 2-3
2. References …………………Pages 4-5

Appendix Table S1

Name	Sequence (5'-3')	Target	Reference
HDV-Ferns-F	GCGCCGGCYGGGCAAC	Total *HDV* RNA	(Ferns *et al*, 2012)
HDV-Ferns-R	TTCCTCTTCGGGTCGGCATG		
HDV-Ferns-Probe	FAM-CGCGGTCCGACCTGGGCATCCG-BHQ1		
Tag1-G-RT Taylor	TCTCCCCAGAGTTGTCGACCCCAGTGAATAAAGCGGGTTTCCACTCACAG	*HDV G*	
Tag1-G-F Taylor	GATTCCTCCCTCTGAGTGCTACT	*HDV G*	
Tag2-AG-RT Taylor	TTCTTCTTTGTCTTCCGGAGGTCTCTCTCGAGTTCCTCTAACTTCTTTCTTCC	*HDV AG*	
Tag2-AG-F Taylor	AGAGGGGACGAGTGAGGCTTATC	*HDV AG*	
AG-R-1	GTCCAGCAGTCTCCTCTTTACAG	*HDV AG*	(Harichandran *et al*, 2019)
HDV (951-970)-F	TATTCACTGGGGTCGACAAC	Total *HDV* RNA	(Verrier *et al*, 2022)
HDV (1082-1101)-R	CTTCGTCCCCAATCTGCAG	Total *HDV* RNA	
HDV-Probe-Non-Edited	FAM-CCTATGGAAATCCCTGGTTTCCCCTGATG-BHQ1	Total *HDV* RNA	
HDV-Probe-Edited	HEX-CCCATGGAAATCCCTGGTTTCCCCTGATG-BHQ1	Total *HDV* RNA	
RPS11-F	GCCGAGACTATCTGCACTAC	*RPS11*	(Dao Thi *et al*, 2020)
RPS11-R	ATGTCCAGCCTCAGAACTTC		
NTCP-F	AAGGACAAGGTGCCCTATAAAGG	*NTCP*	
NTCP-R	TTGAGGACGATCCCTATGGTG		
AFP-F	TGGGACCCGAACTTTCCA	*AFP*	
AFP-R	GGCCACATCCAGGACTAGTTTC		
ALB-F	GGTGTTGATTGCCTTTGCTC	*ALB*	
ALB-R	CCCTTCATCCCGAAGTTCAT		
CAV1-F	GCGACCCTAAACACCTCAAC	*CAV1*	(Spandidos *et al*, 2009)
CAV1-R	ATGCCGTCAAAACTGTGTGTC		
CAV2-F	AAGACCTGCCTAATGGTTCTGC	*CAV2*	
CAV2-R	CTCGTACACAATGGAGCAATGAT		
EGFR-F	AGGCACGAGTAACAAGCTCAC	*EGFR*	
EGFR-R	ATGAGGACATAACCAGCCACC		
LAMP1-F	TCTCAGTGAACTACGACACCA	*LAMP1*	
LAMP1-R	AGTGTATGTCCTCTTCCAAAAGC		
SCARB1-F	CCTATCCCCTTCTATCTCTCCG	*SCARB1*	
SCARB1-R	GGATGTTGGGCATGACGATGT		
SCARB2-F	AGATGGAGATTCTTTTCACCCAC	*SCARB2*	
SCARB2-R	CAGGAACTTTATACCGAAAGGCA		
ITGA2-F	CCTACAATGTTGGTCTCCCAGA	*ITGα1*	
ITGA2-R	AGTAACCAGTTGCCTTTTGGATT		

ITGB1-F	CCTACTTCTGCACGATGTGATG	*ITGβ1*	
ITGB1-R	CCTTTGCTACGGTTGGTTACATT		
LDLR-F	TCTGCAACATGGCTAGAGACT	*LDLR*	
LDLR-R	TCCAAGCATTCGTTGGTCCC		
SDC2-F	AAATGGACCCAGCCGAAGAG	*SDC2*	
SDC2-R	TGGTTTGCGTTCTCCAAGGT		
GPC5-F	AGACGCTTGCCAACAGAAGA	*GPC5*	
GPC5-R	CCATTTCCAACCACACGCTG		
POLR2A-F	CCCCAACCTCTCCATTGACC	*POLR2A*	
POLR2A-R	CCCTGCGCACTAGTTCTTGA		
CAD-F	GCGGTGCTGCTATGAATGTG	*CAD*	
CAD-R	ATGCTCAGAGATGGCGATGG		
MOV10-F	TGCTCCCCATGCTTCTTCAG	*MOV10*	
MOV10-R	GTGCCGGATATCATGCTCCA		
CD63-F	CAGTGGTCATCATCGCAGTG	*CD63*	
CD63-R	ATCGAAGCAGTGTGGTTGTTT		
HBV cccDNA-F	CCGTGTGCACTTCGCTTCA	*HBV* cccDNA	(Allweiss *et al*, 2023)
HBV cccDNA-R	GCACAGCTTGGAGGCTTGA		
HBV cccDNA-probe	FAM-CATGGAGACCACCGTGAACGCCC-BHQ1		
HBV-F	GTTGCCCGTTTGTCCTCTAATTC	Total *HBV* DNA	(Qu *et al*, 2018)
HBV-R	GGAGGGATACATAGAGGTTCCTTGA		
β-globin-F	AGGTACGGCTGTCATCACTTAGA	*β-globin*	
β-globin-R	CATGGTGTCTGTTTGAGGTTGCTA		

References

Alexa A, Rahnenfuhrer J (2016) topGO: enrichment analysis for Gene Ontology. R Packag. version 2.26.0. *R Package version 2260*

Allweiss L, Testoni B, Yu M, Lucifora J, Ko C, Qu B, Lütgehetmann M, Guo H, Urban S, Fletcher SP (2023) Quantification of the hepatitis B virus cccDNA: evidence-based guidelines for monitoring the key obstacle of HBV cure. *Gut* 72: 972-983

Börner K, Kienle E, Huang LY, Weinmann J, Sacher A, Bayer P, Stüllein C, Fakhiri J, Zimmermann L, Westhaus A *et al* (2020) Pre-arrayed Pan-AAV Peptide Display Libraries for Rapid Single-Round Screening. *Molecular Therapy* 28: 1016-1032

Dao Thi VL, Wu X, Belote RL, Andreo U, Takacs CN, Fernandez JP, Vale-Silva LA, Prallet S, Decker CC, Fu RM *et al* (2020) Stem cell-derived polarized hepatocytes. *Nature Communications* 11: 1677-1677

Ferns RB, Nastouli E, Garson JA (2012) Quantitation of hepatitis delta virus using a single-step internally controlled real-time RT-qPCR and a full-length genomic RNA calibration standard. *Journal of Virological Methods* 179: 189-194

Gripon P, Rumin S, Urban S, Le Seyec J, Glaise D, Cannie I, Guyomard C, Lucas J, Trepo C, Guguen-Guillouzo C (2002) Infection of a human hepatoma cell line by hepatitis B virus. *Proceedings of the National Academy of Sciences of the United States of America* 99: 15655-15660

Gudima S, Chang J, Moraleda G, Azvolinsky A, Taylor J (2002) Parameters of Human Hepatitis Delta Virus Genome Replication: the Quantity, Quality, and Intracellular Distribution of Viral Proteins and RNA. *Journal of Virology* 76: 3709-3719

Harichandran K, Shen Y, Stephenson Tsoris S, Lee S-C, Casey JL (2019) Hepatitis delta antigen regulates mRNA and antigenome RNA levels during hepatitis delta virus replication. *Journal of Virology* 93: 10.1128/jvi. 01989-01918

Lempp FA, Schlund F, Rieble L, Nussbaum L, Link C, Zhang Z, Ni Y, Urban S (2019) Recapitulation of HDV infection in a fully permissive hepatoma cell line allows efficient drug evaluation. *Nature Communications* 10: 1-11

Love MI, Huber W, Anders S (2014) Moderated estimation of fold change and dispersion for RNA-seq data with DESeq2. *Genome Biology* 15: 1-21

Maurer L, Andari JE, Rapti K, Spreyer L, Steinmann E, Grimm D, Thi VLD (2022) Induction of Hepatitis E Virus Anti-ORF3 Antibodies from Systemic Administration of a Muscle-Specific Adeno-Associated Virus (AAV) Vector. *Viruses* 14: 266-266

Ni Y, Lempp FA, Mehrle S, Nkongolo S, Kaufman C, Fälth M, Stindt J, Königer C, Nassal M, Kubitz R *et al* (2014) Hepatitis B and D viruses exploit sodium taurocholate co-transporting polypeptide for species-specific entry into hepatocytes. *Gastroenterology* 146: 1070-1083

Patro R, Duggal G, Love MI, Irizarry RA, Kingsford C (2017) Salmon provides fast and bias-aware quantification of transcript expression. *Nature Methods* 14: 417-419

Qu B, Ni Y, Lempp FA, Vondran FW, Urban S (2018) T5 exonuclease hydrolysis of hepatitis B virus replicative intermediates allows reliable quantification and fast drug efficacy testing of covalently closed circular DNA by PCR. *Journal of virology* 92: 10.1128/jvi. 01117-01118

Soneson C, Love MI, Robinson MD (2015) Differential analyses for RNA-seq: transcript-level estimates improve gene-level inferences. *F1000Research* 4

Spandidos A, Wang X, Wang H, Seed B (2009) PrimerBank: A resource of human and mouse PCR primer pairs for gene expression detection and quantification. *Nucleic Acids Research* 38: D792-D799

Thomson JA, Itskovitz-Eldor J, Shapiro SS, Waknitz MA, Swiergiel JJ, Marshall VS, Jones JM (1998) Embryonic stem cell lines derived from human blastocysts. *science* 282: 1145-1147

Verrier ER, Salvetti A, Pons C, Michelet M, Rivoire M, Baumert TF, Durantel D, Lucifora J (2022) Loss of hepatitis D virus infectivity upon farnesyl transferase inhibitor treatment associates with increasing RNA editing rates revealed by a new RT-ddPCR method. *Antiviral Research* 198: 105250

Wang W, Lempp FA, Schlund F, Walter L, Decker CC, Zhang Z, Ni Y, Urban S (2021) Assembly and infection efficacy of hepatitis B virus surface protein exchanges in 8 hepatitis D virus genotype isolates. *Journal of Hepatology* 75: 311-323
Wolff G, Taranko AE, Meln I, Weinmann J, Sijmonsma T, Lerch S, Heide D, Billeter AT, Tews D, Krunic D *et al* (2019) Diet-dependent function of the extracellular matrix proteoglycan Lumican in obesity and glucose homeostasis. *Molecular Metabolism* 19: 97-106
