# Supplementary figures and images for: An hepatitis B and D virus infection model using human pluripotent stem cell-derived hepatocytes
Source: EMBO Rep. 2024 Sep 4;25(10):17. doi: 10.1038/s44319-024-00236-0 (PMC11466959; doi:10.1038/s44319-024-00236-0)

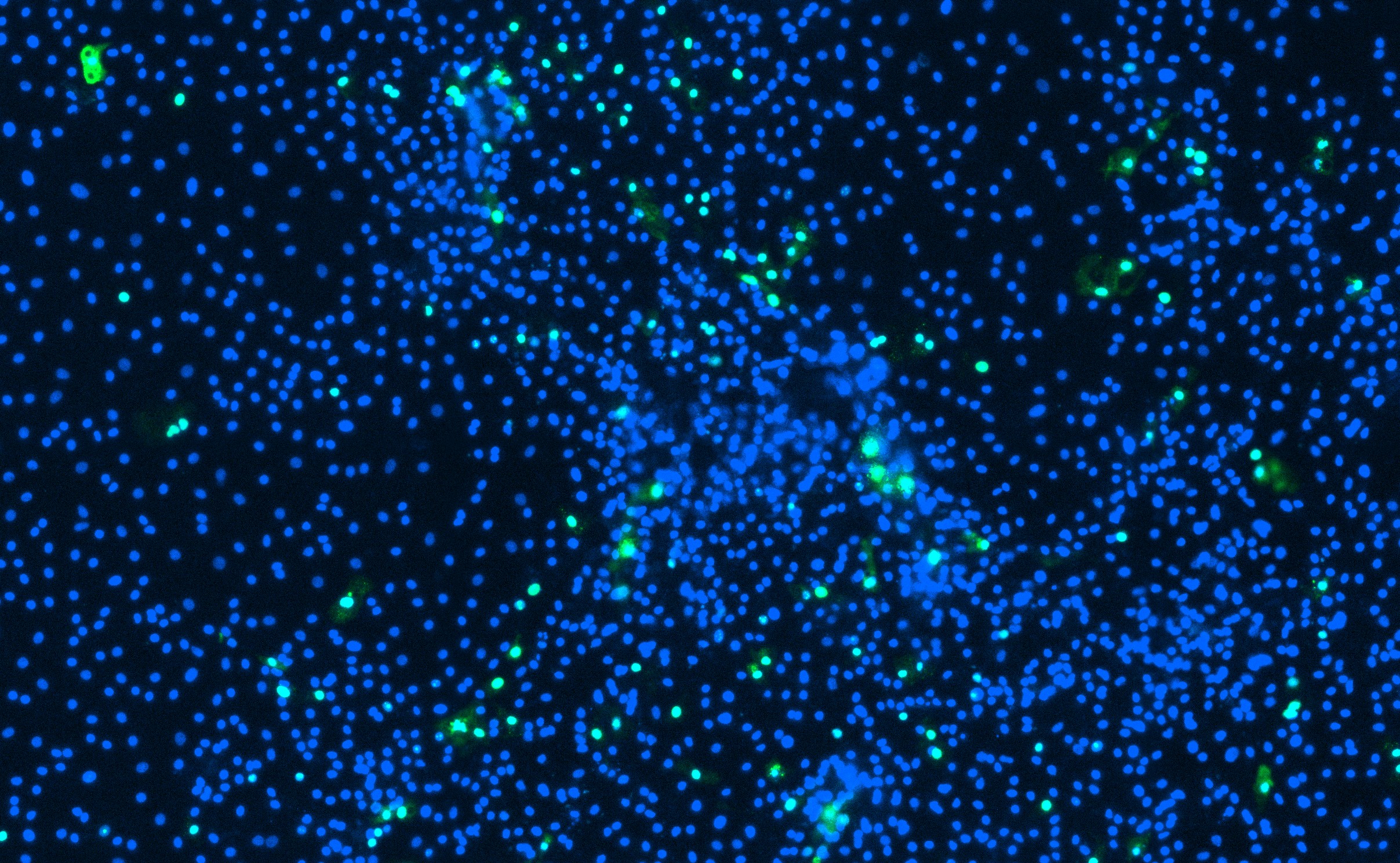

Supplement: Supplementary file 3 — Source data Fig. 1 [file 44319_2024_236_MOESM3_ESM.zip › 1A/HDV GT1T/HDV GT1T_merge_HDAg(green)+nuclei.jpeg]

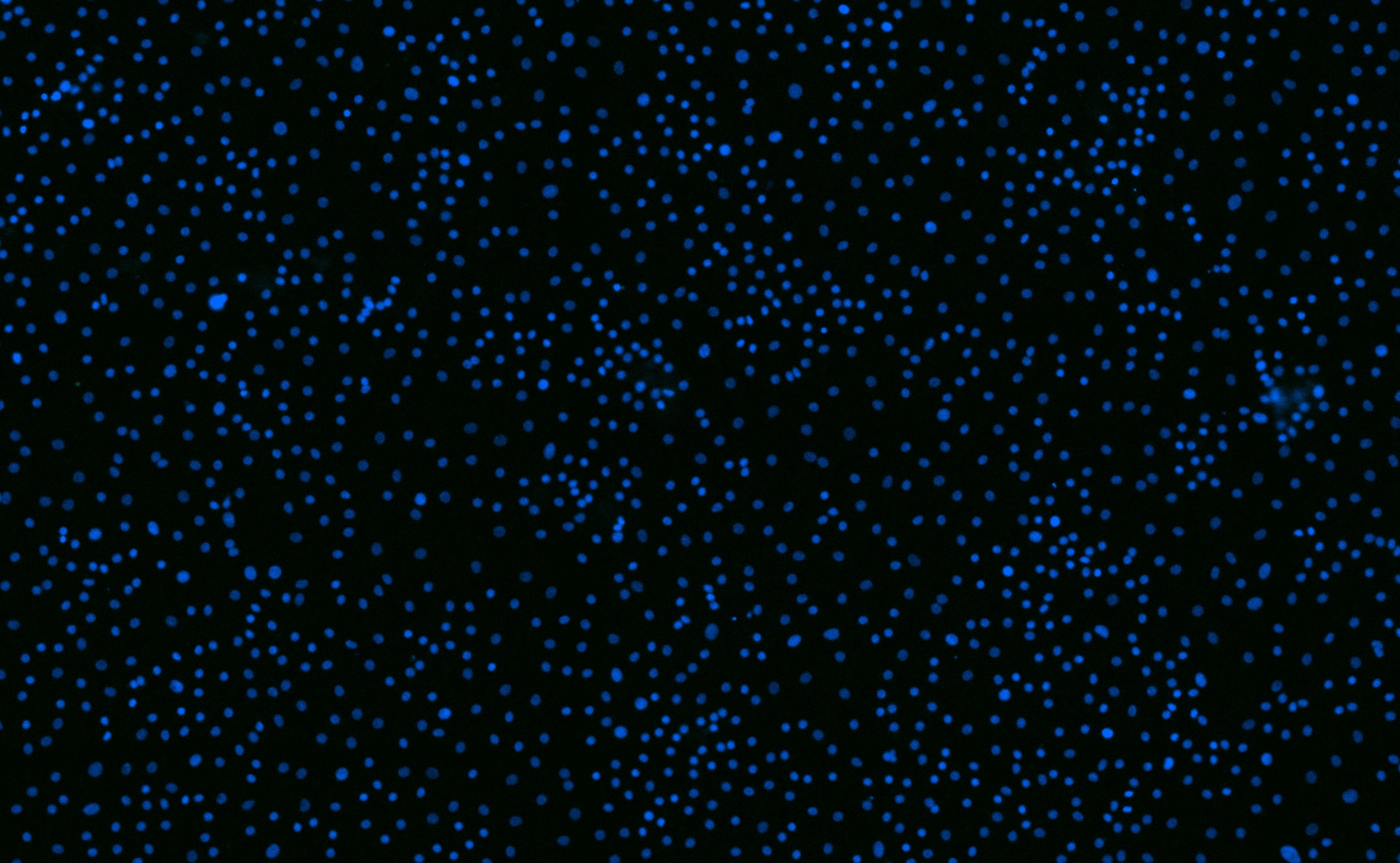

Supplement: Supplementary file 3 — Source data Fig. 1 [file 44319_2024_236_MOESM3_ESM.zip › 1A/HDV GT1T+BLV/HDV GT1T+BLV_merge_HDAg(green)+nuclei.jpeg]

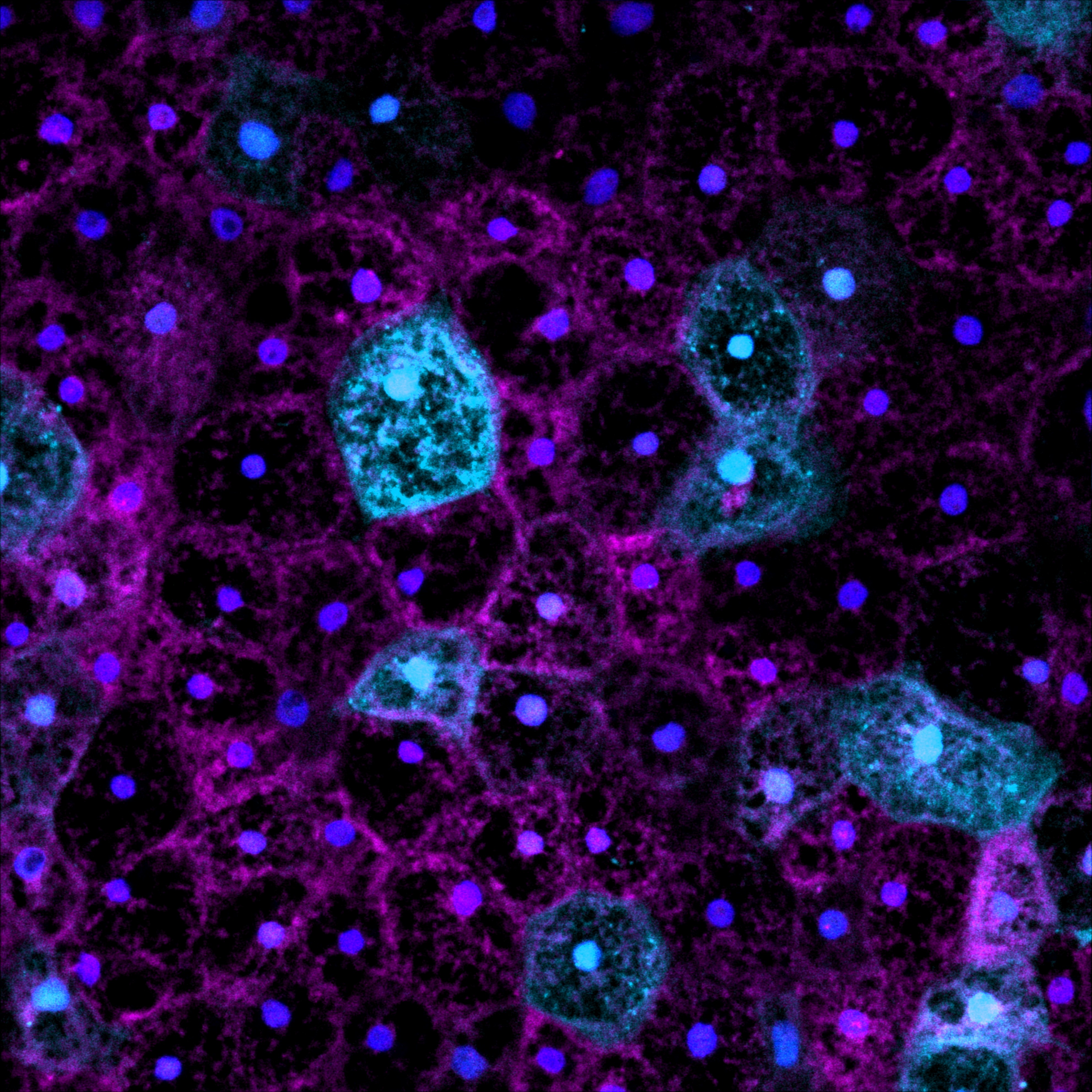

Supplement: Supplementary file 3 — Source data Fig. 1 [file 44319_2024_236_MOESM3_ESM.zip › 1D/Fig 1D (Left)_HLC transduced with AAV-YFP/Fig 1D (Left)_HLC transduced with AAV-YFP_overlay.jpeg]

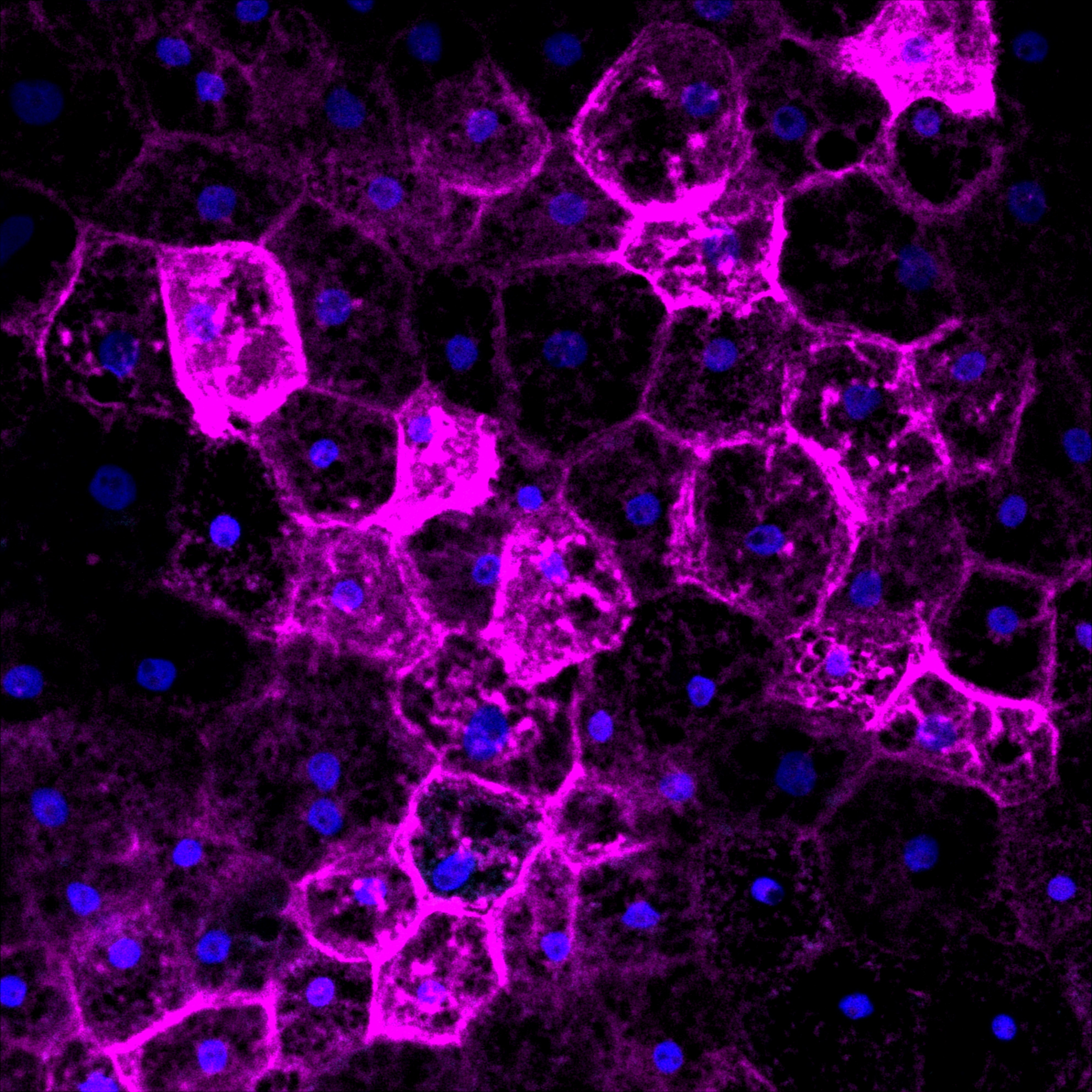

Supplement: Supplementary file 3 — Source data Fig. 1 [file 44319_2024_236_MOESM3_ESM.zip › 1D/Fig 1D (Right)_HLC transduced with AAV-NTCP/Fig 1D (Right)_HLC transduced with AAV-NTCP_overlay.jpeg]

## Slide 1
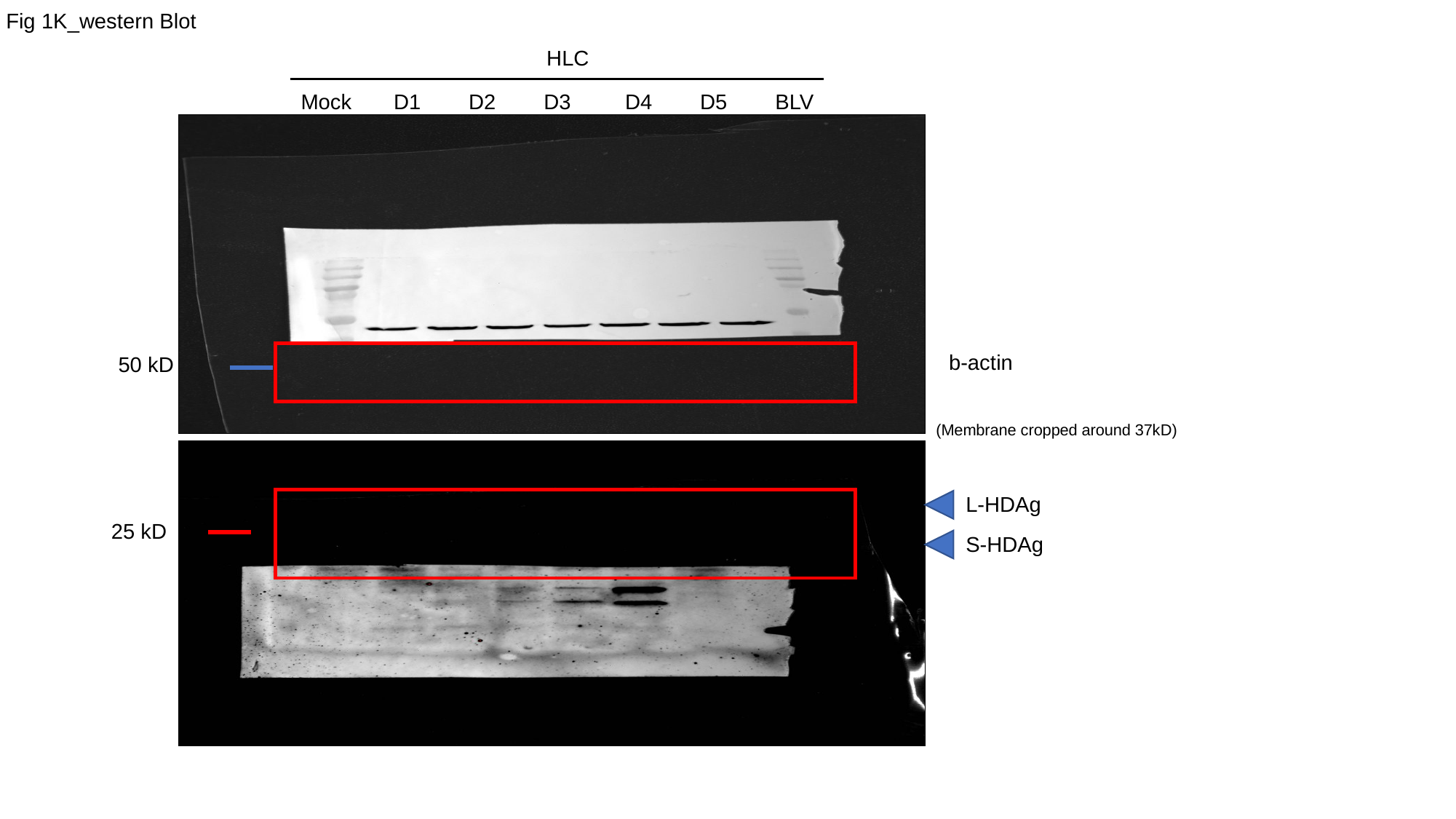

Fig 1K_western Blot
HLC
Mock D1 D2 D3 D4 D5 BLV
b-actin
50 kD
(Membrane cropped around 37kD)
L-HDAg
25 kD
S-HDAg

Supplement: Supplementary file 3 — Source data Fig. 1 [file 44319_2024_236_MOESM3_ESM.zip › 1K/Fig 1K.pptx]

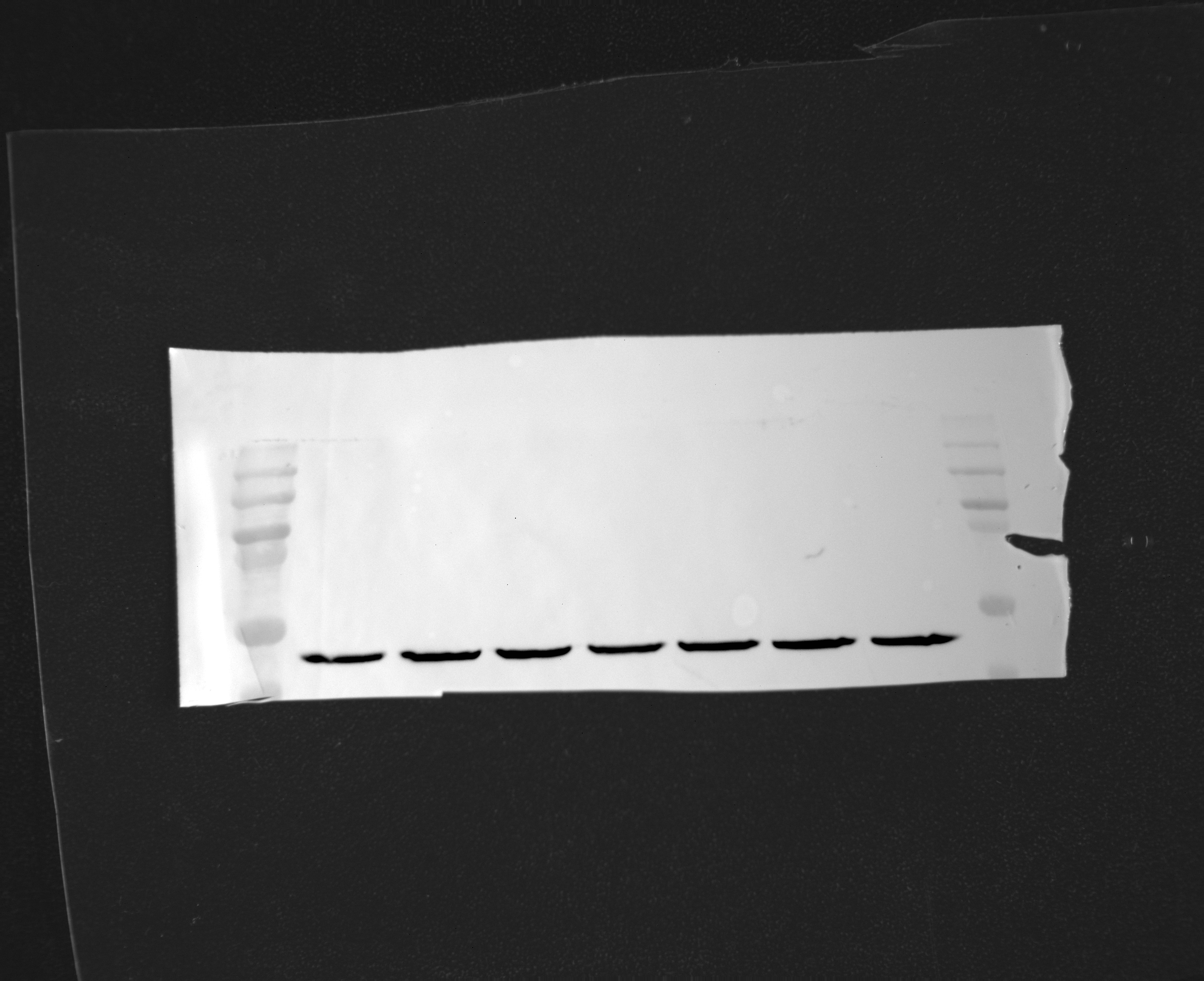

Supplement: Supplementary file 3 — Source data Fig. 1 [file 44319_2024_236_MOESM3_ESM.zip › 1K/Fig 1K_bactin/Fig 1K_bactin_ scan+Marker_image.tif]

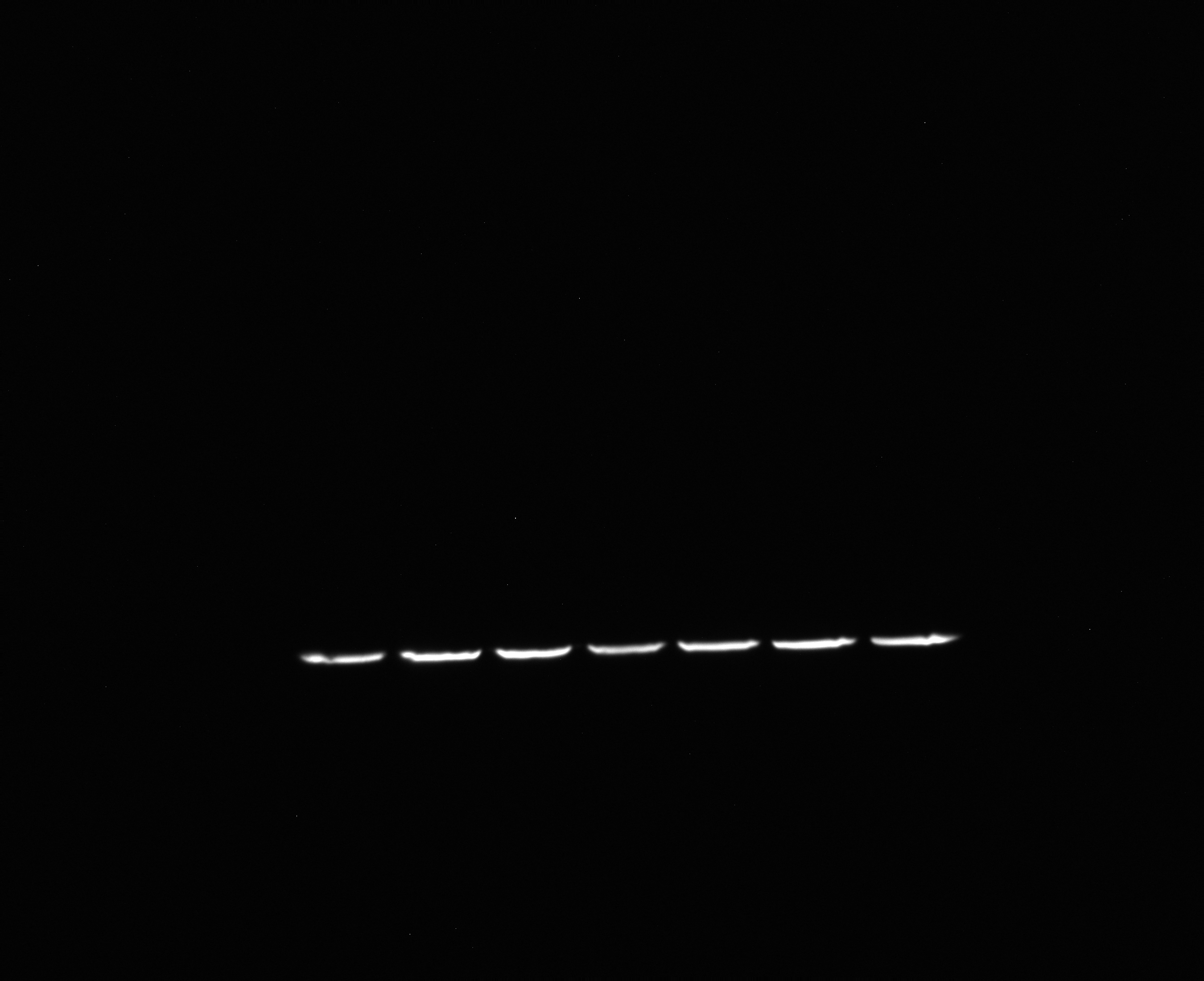

Supplement: Supplementary file 3 — Source data Fig. 1 [file 44319_2024_236_MOESM3_ESM.zip › 1K/Fig 1K_bactin/Fig 1K_bactin_raw data.tif]

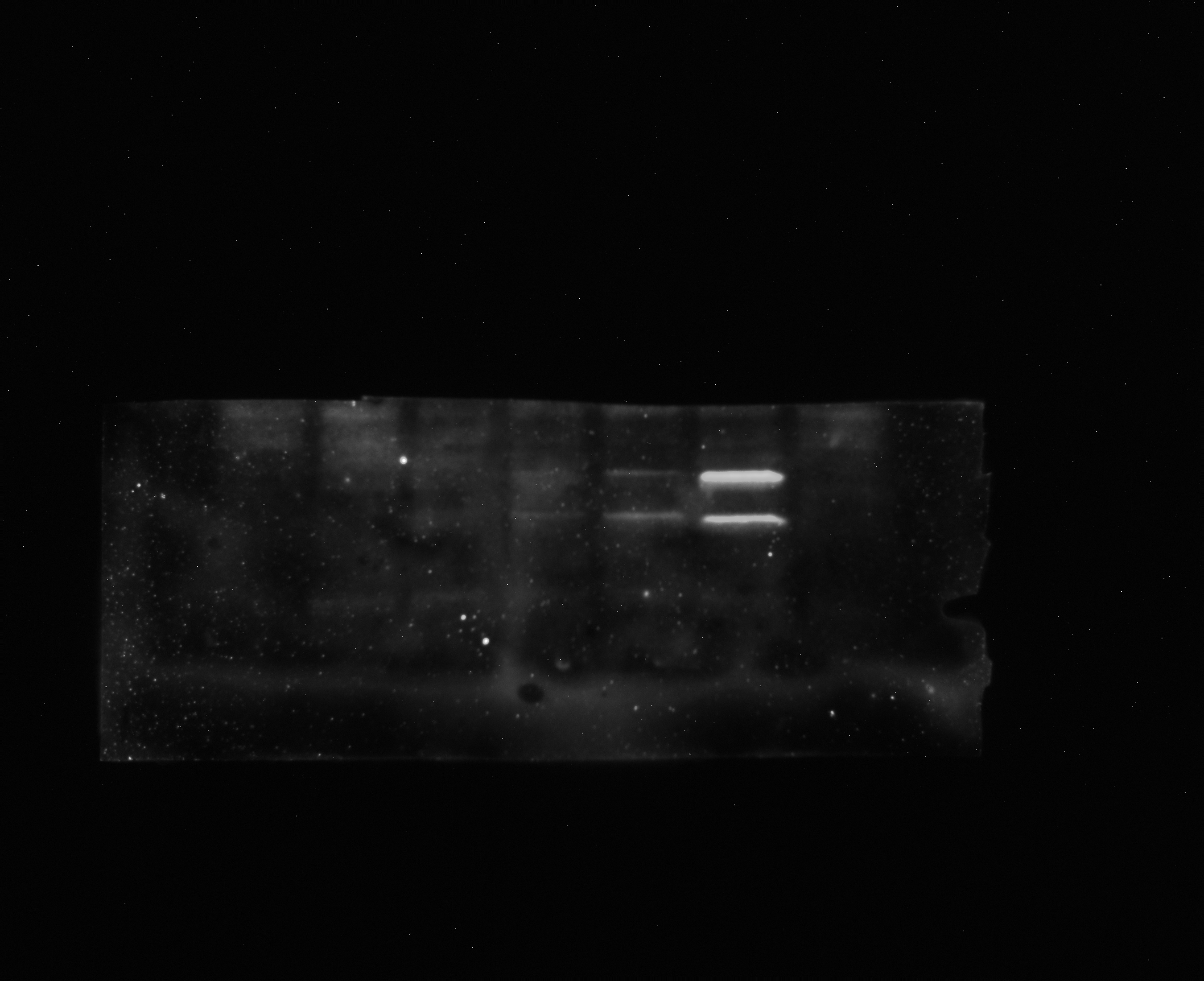

Supplement: Supplementary file 3 — Source data Fig. 1 [file 44319_2024_236_MOESM3_ESM.zip › 1K/Fig 1K_HDAg/Fig 1K_HDAg_ raw data.tif]

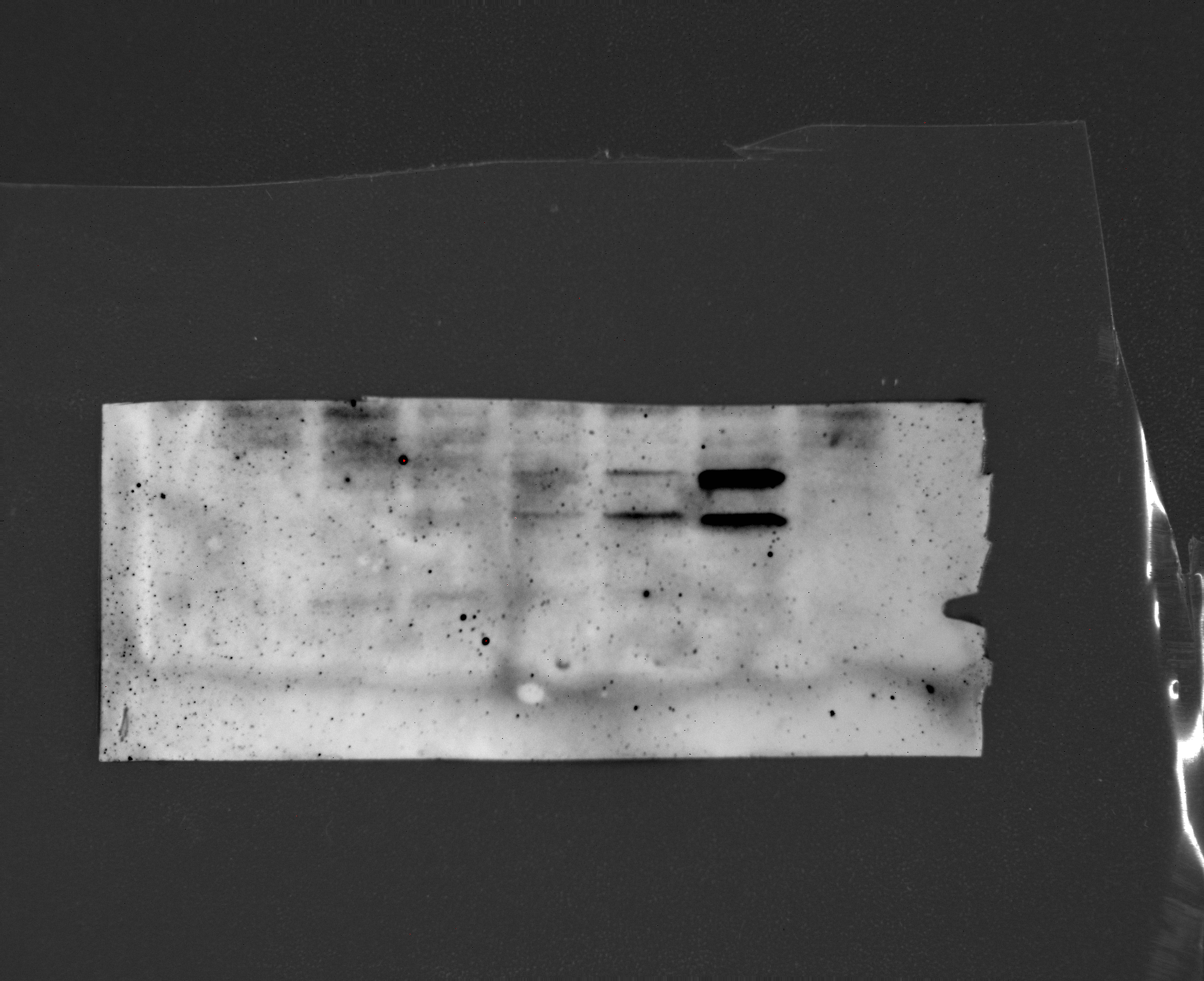

Supplement: Supplementary file 3 — Source data Fig. 1 [file 44319_2024_236_MOESM3_ESM.zip › 1K/Fig 1K_HDAg/Fig 1K_HDAg_ scan+Marker_image.tif]

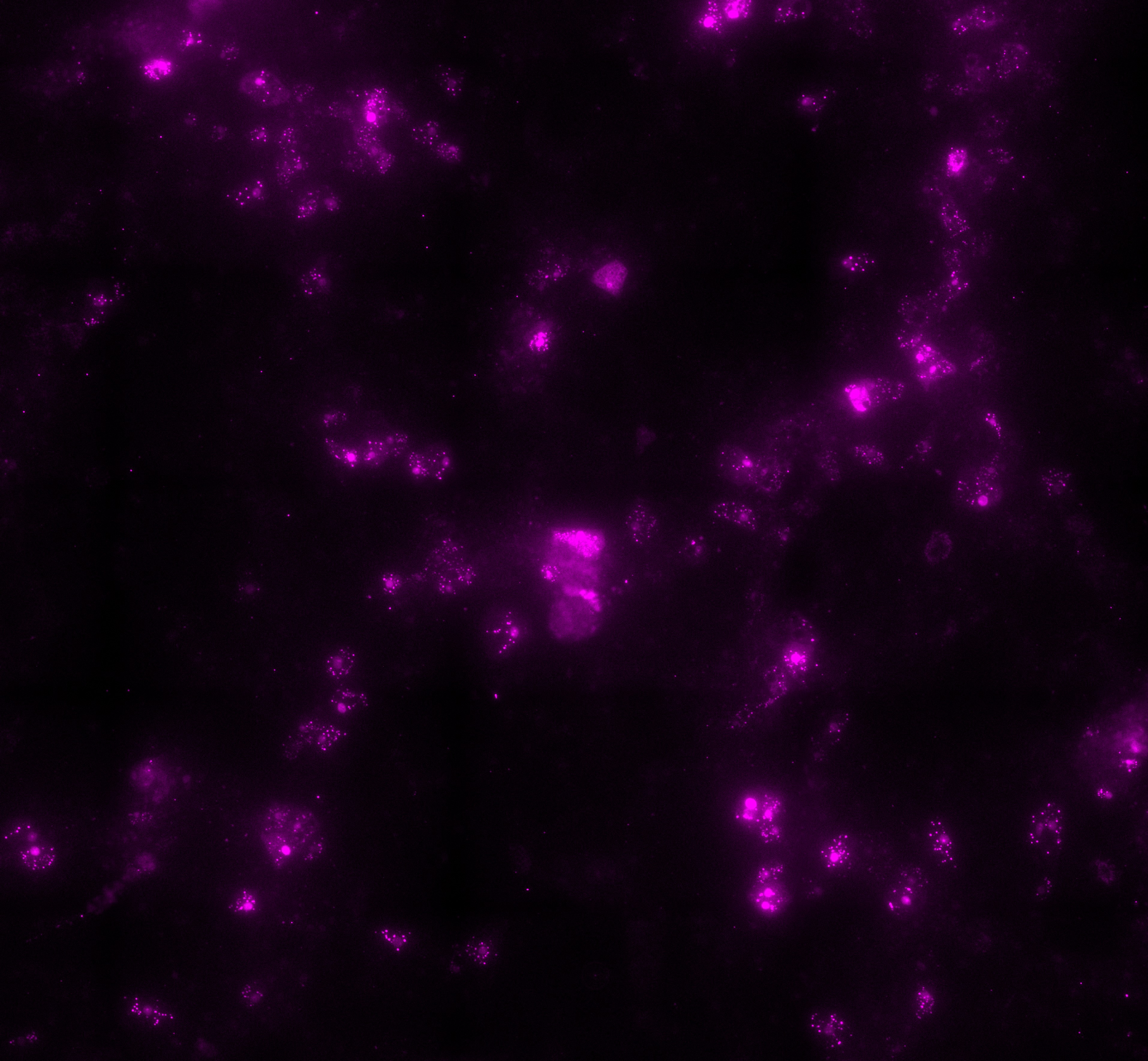

Supplement: Supplementary file 4 — Source data Fig. 2 [file 44319_2024_236_MOESM4_ESM.zip › 2A/Fig 2A_HBcAg.jpeg]

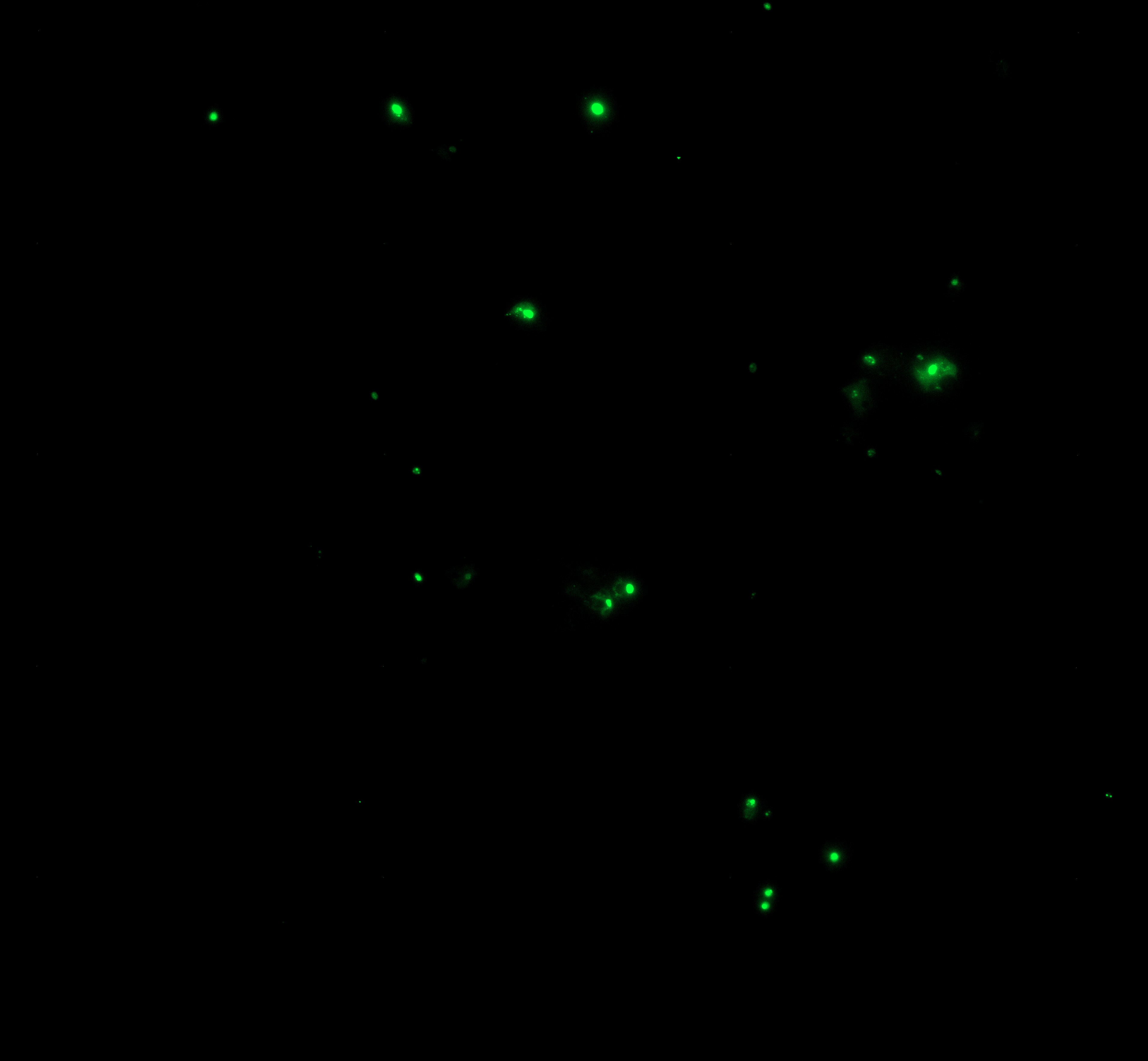

Supplement: Supplementary file 4 — Source data Fig. 2 [file 44319_2024_236_MOESM4_ESM.zip › 2A/Fig 2A_HDAg.jpeg]

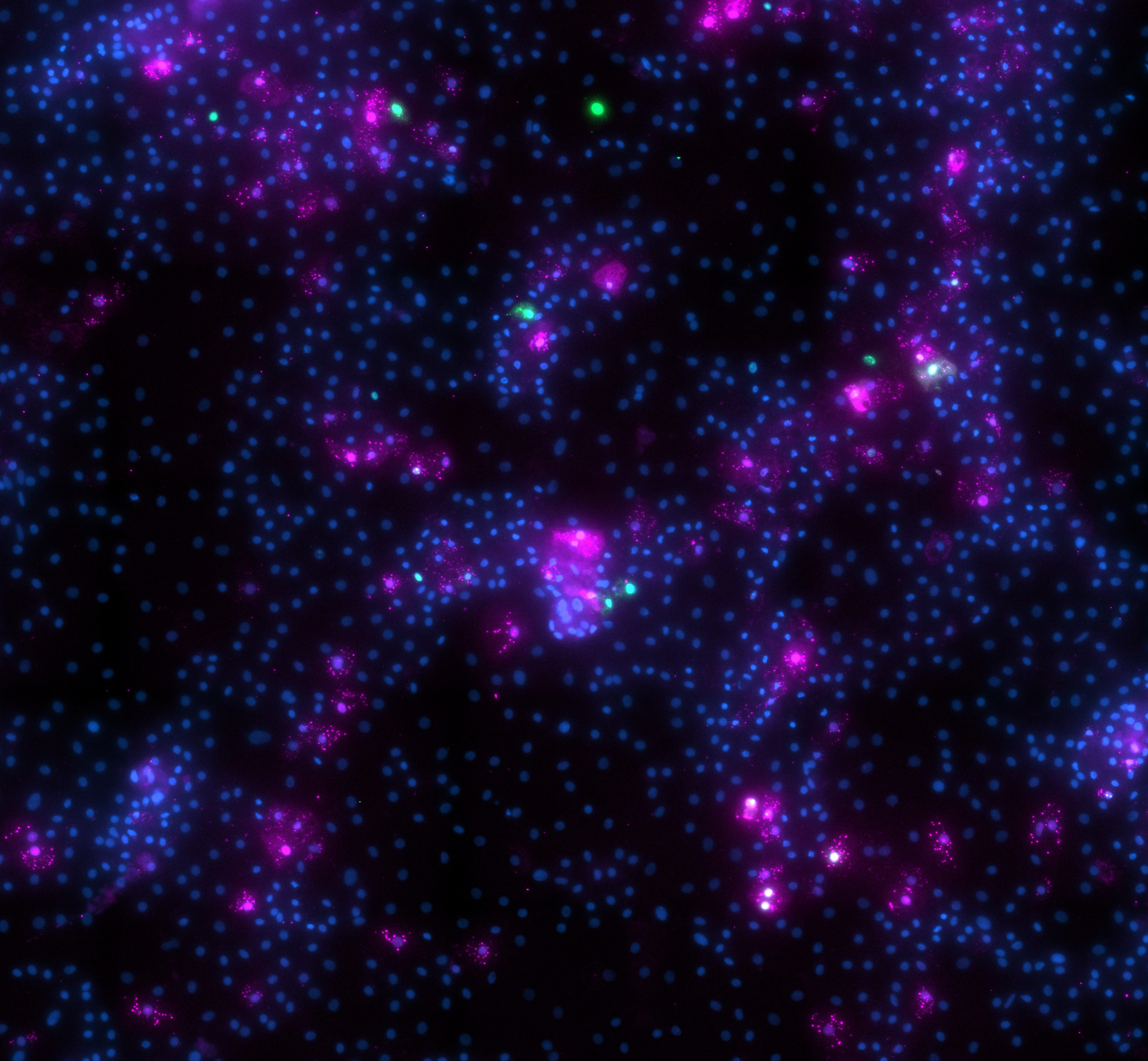

Supplement: Supplementary file 4 — Source data Fig. 2 [file 44319_2024_236_MOESM4_ESM.zip › 2A/Fig 2A_overlay.jpeg]

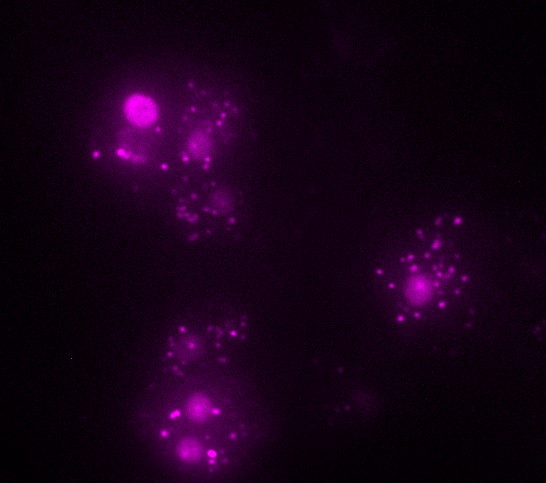

Supplement: Supplementary file 4 — Source data Fig. 2 [file 44319_2024_236_MOESM4_ESM.zip › 2A/Fig 2A_zoom in_HBcAg.jpeg]

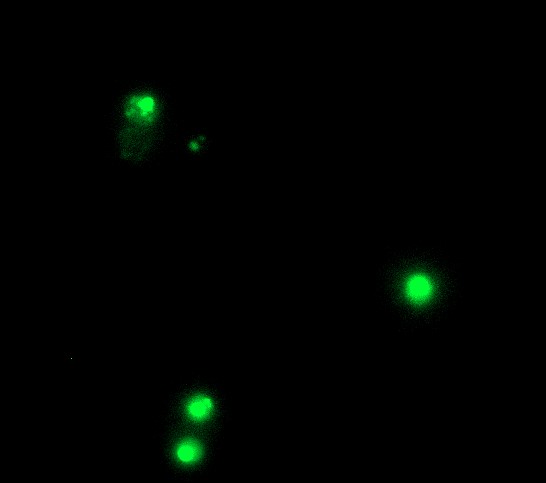

Supplement: Supplementary file 4 — Source data Fig. 2 [file 44319_2024_236_MOESM4_ESM.zip › 2A/Fig 2A_zoom in_HDAg.jpeg]

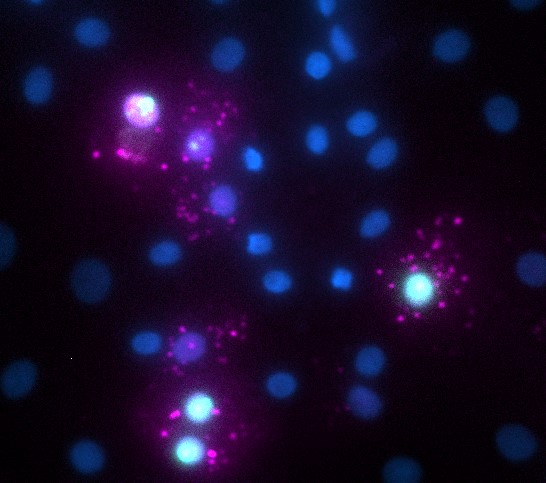

Supplement: Supplementary file 4 — Source data Fig. 2 [file 44319_2024_236_MOESM4_ESM.zip › 2A/Fig 2A_zoom in_overlay.jpeg]

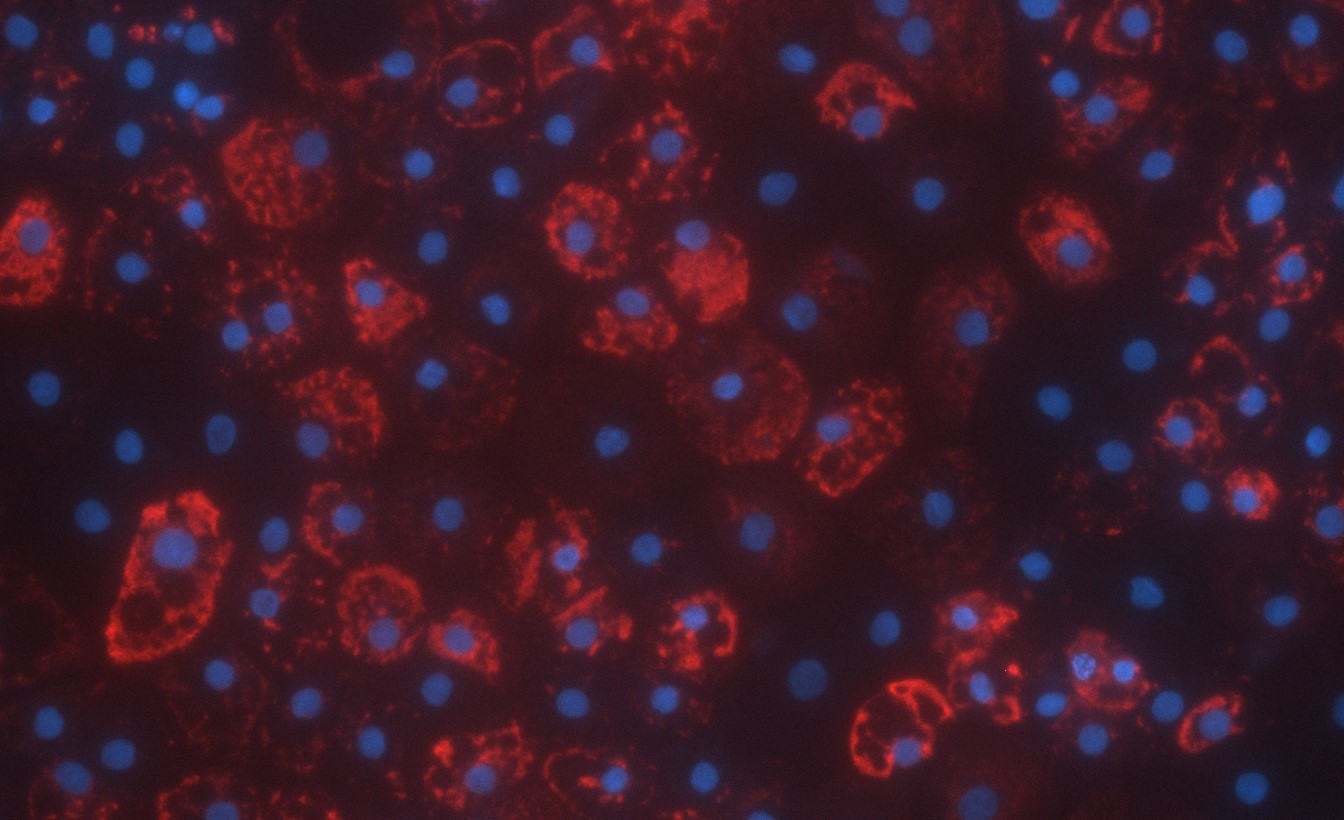

Supplement: Supplementary file 5 — Source data Fig. 3 [file 44319_2024_236_MOESM5_ESM.zip › 3A/AAV-HBsAg/AAV-HBsAg_merge_HBsAg+nuclei.jpeg]

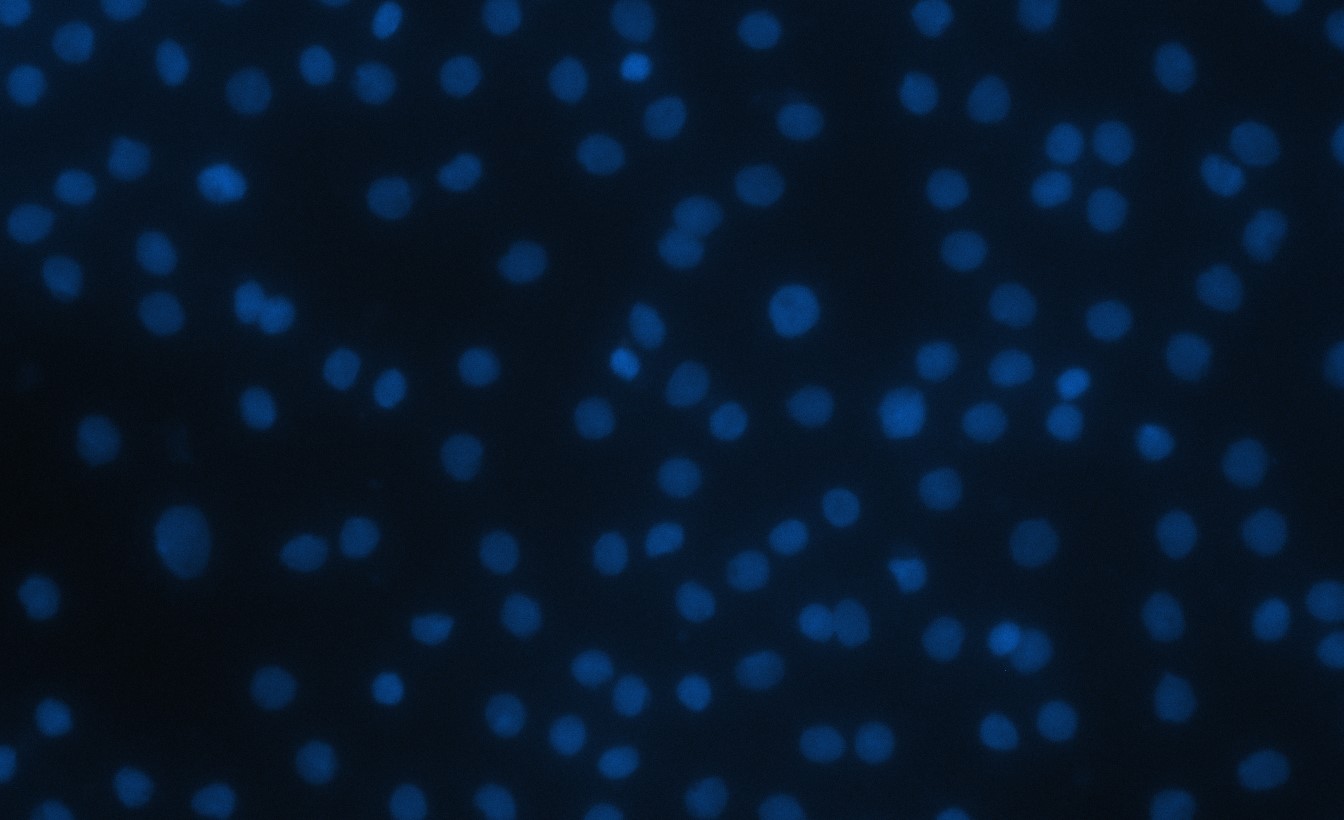

Supplement: Supplementary file 5 — Source data Fig. 3 [file 44319_2024_236_MOESM5_ESM.zip › 3A/AAV-YFP/AAV-YFP_merge_HBsAg+nuclei.jpeg]

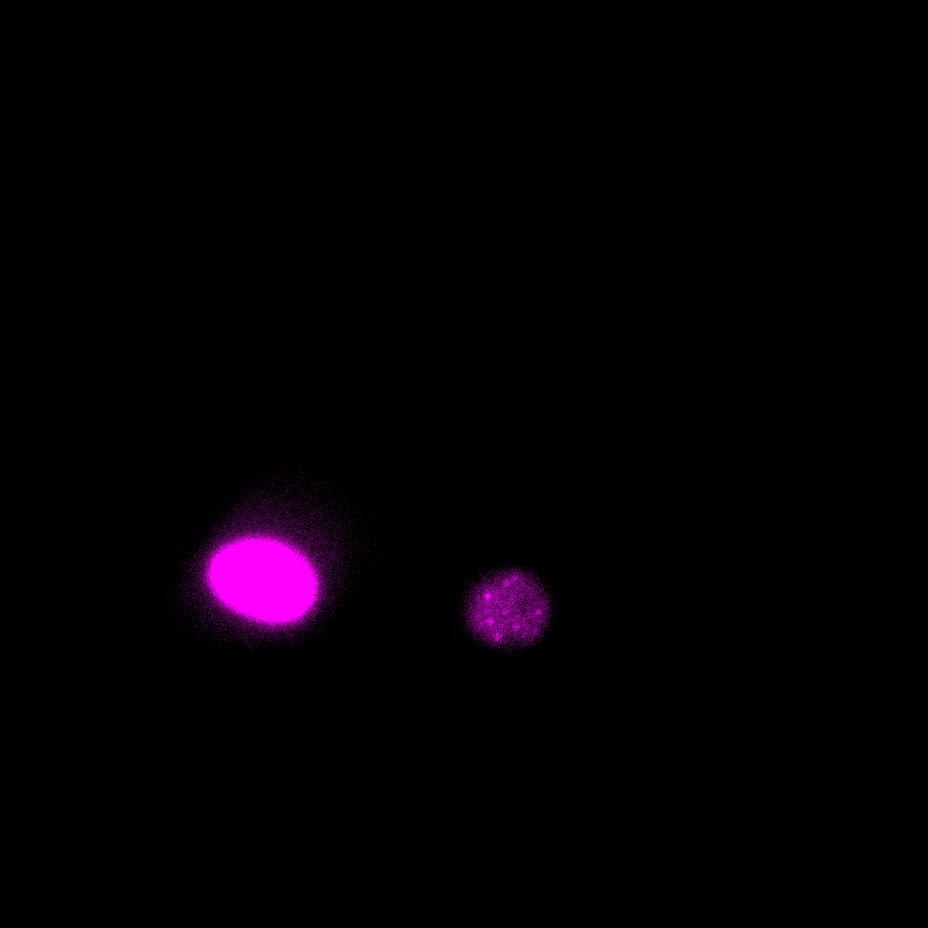

Supplement: Supplementary file 5 — Source data Fig. 3 [file 44319_2024_236_MOESM5_ESM.zip › 3F/HDV/HDAg-magenta.jpeg]

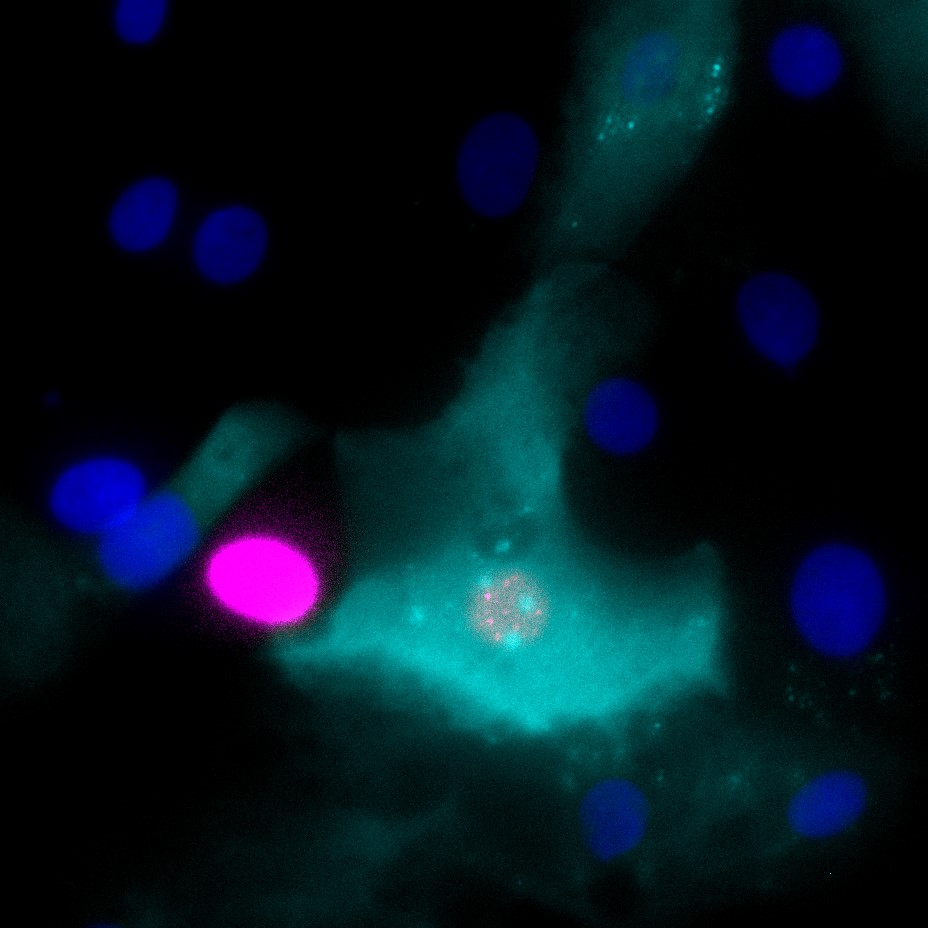

Supplement: Supplementary file 5 — Source data Fig. 3 [file 44319_2024_236_MOESM5_ESM.zip › 3F/HDV/Merge_HDAg+Zsgreen+nuclei.jpeg]

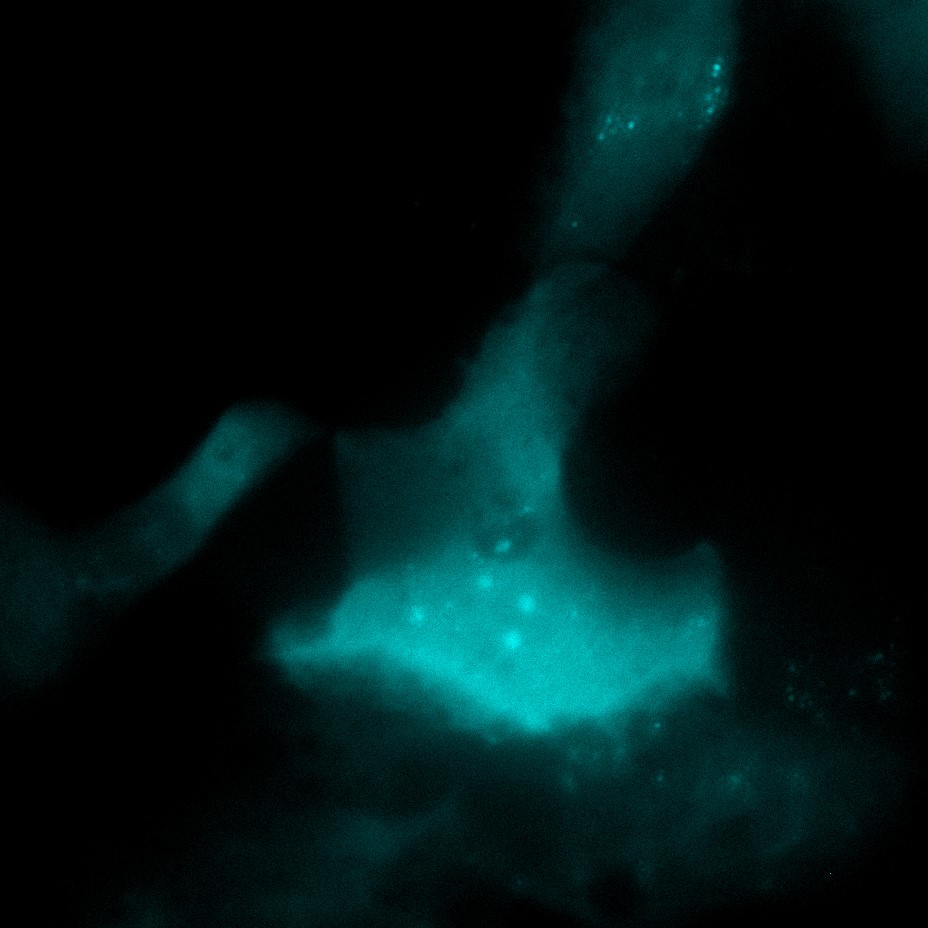

Supplement: Supplementary file 5 — Source data Fig. 3 [file 44319_2024_236_MOESM5_ESM.zip › 3F/HDV/Zs-green_Cyan.jpeg]

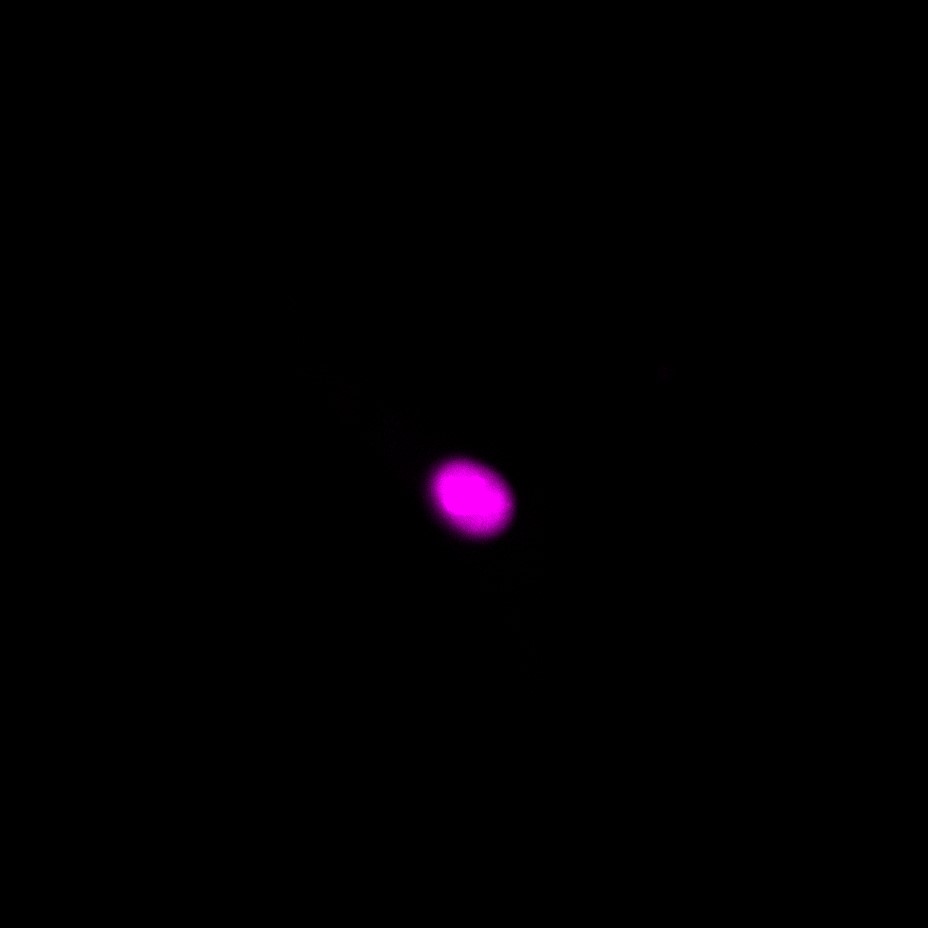

Supplement: Supplementary file 5 — Source data Fig. 3 [file 44319_2024_236_MOESM5_ESM.zip › 3F/HDV+BLV/HDAg_mengenta.jpeg]

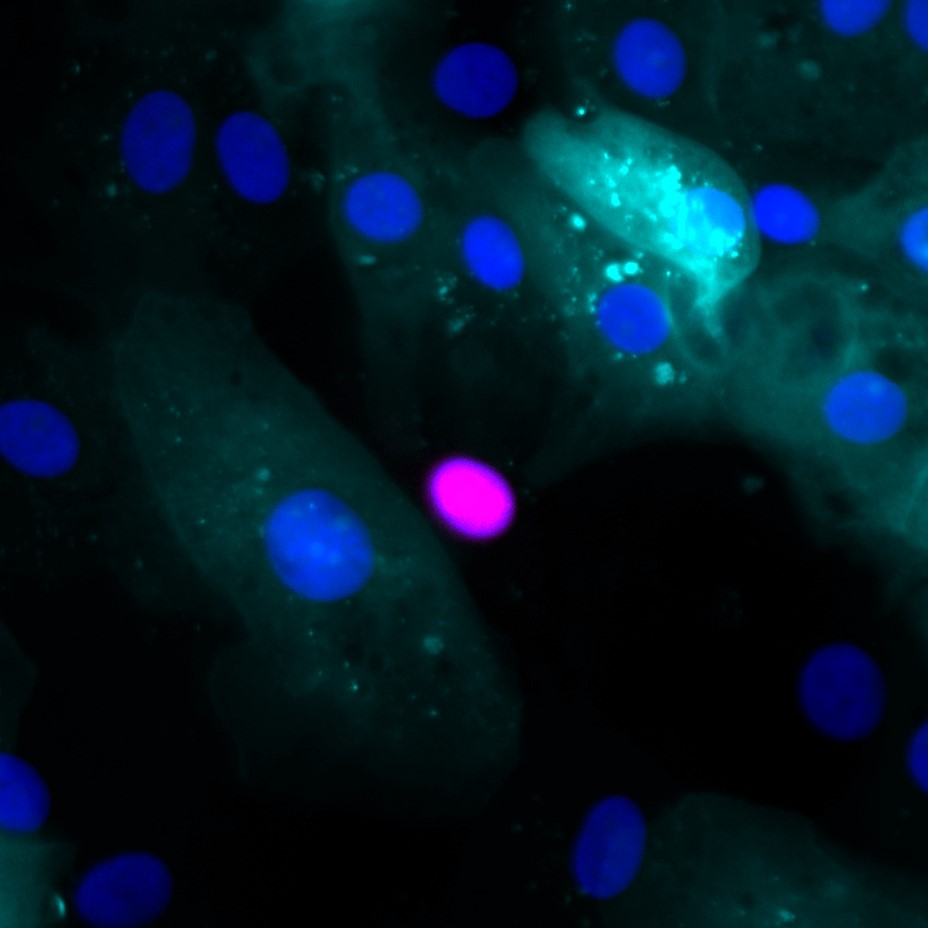

Supplement: Supplementary file 5 — Source data Fig. 3 [file 44319_2024_236_MOESM5_ESM.zip › 3F/HDV+BLV/Merge_HDAg+Zs green+nuclei.jpeg]

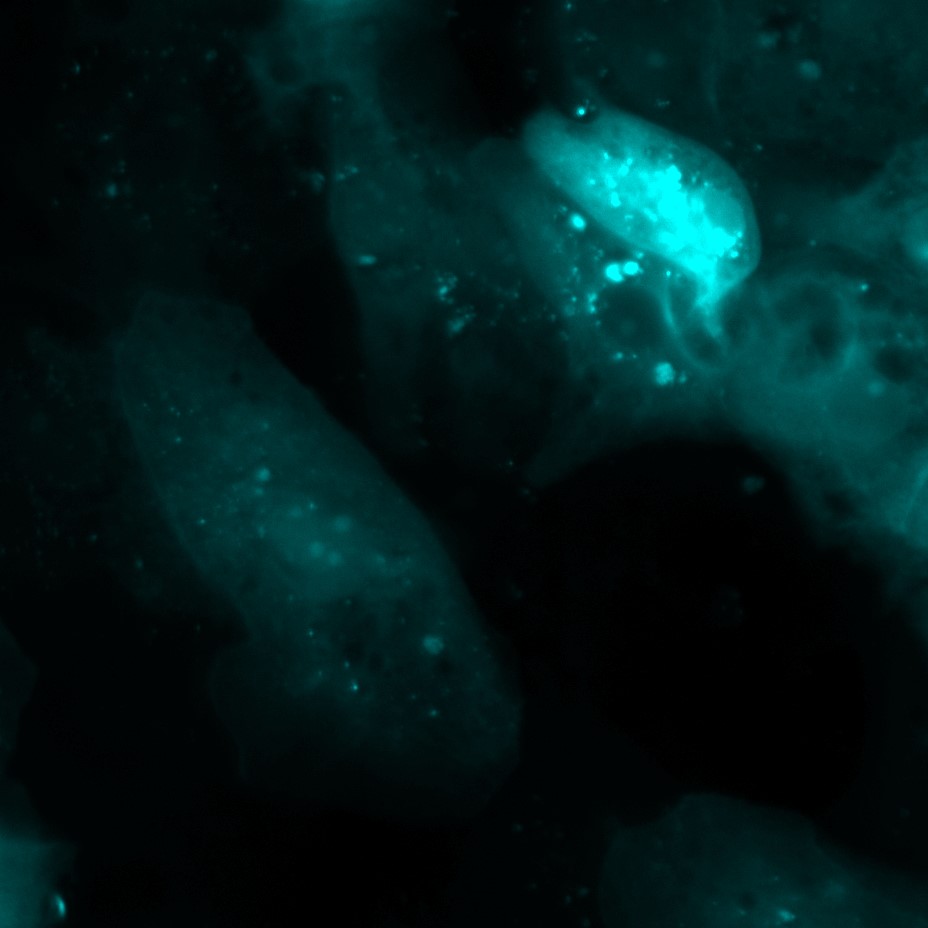

Supplement: Supplementary file 5 — Source data Fig. 3 [file 44319_2024_236_MOESM5_ESM.zip › 3F/HDV+BLV/Zs-green_Cyan.jpeg]

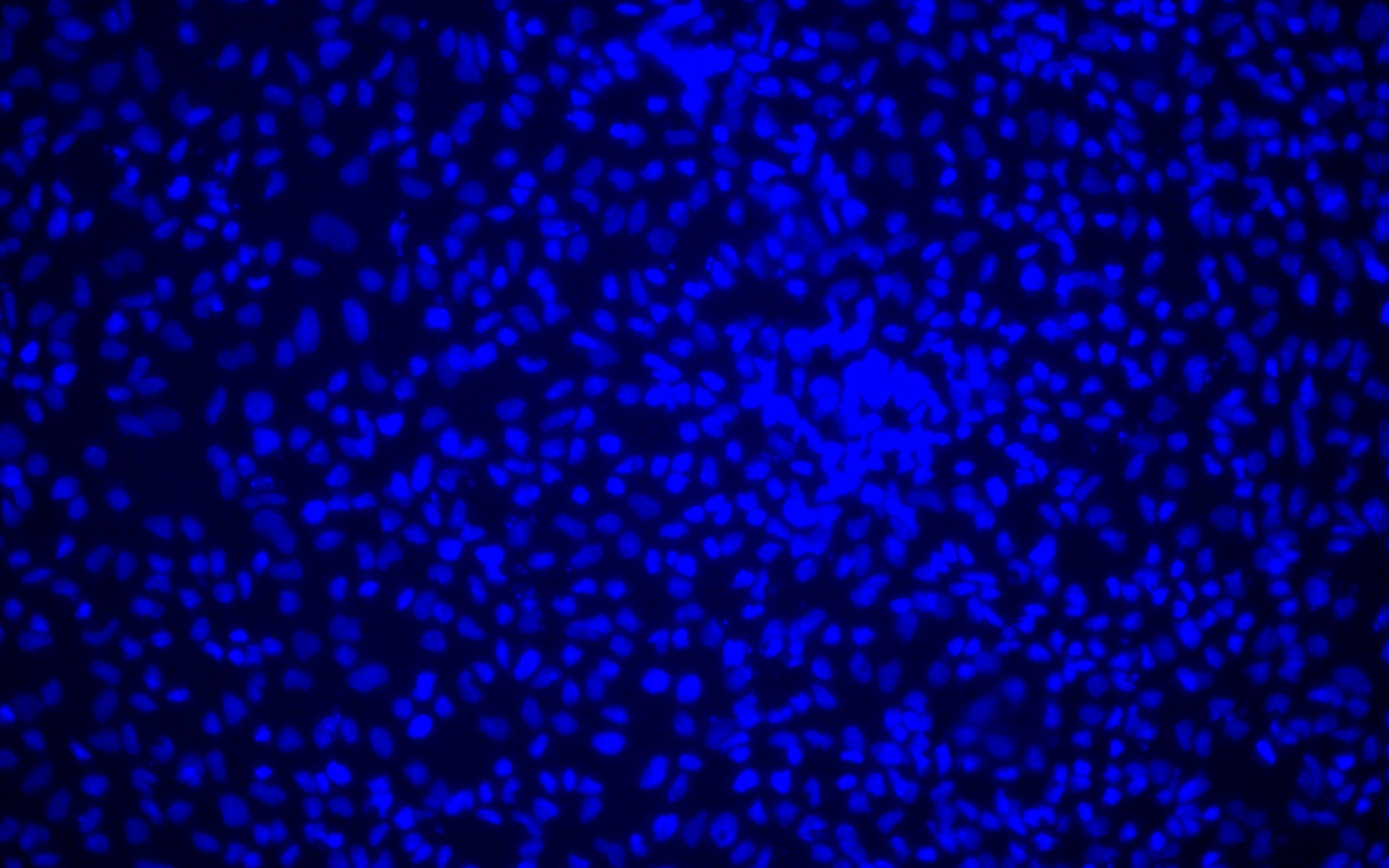

Supplement: Supplementary file 6 — Source data Fig. 4 [file 44319_2024_236_MOESM6_ESM.zip › Fig 4A/AFP/definite endoderm/Merge_AFP (red)+nuclei.jpeg]

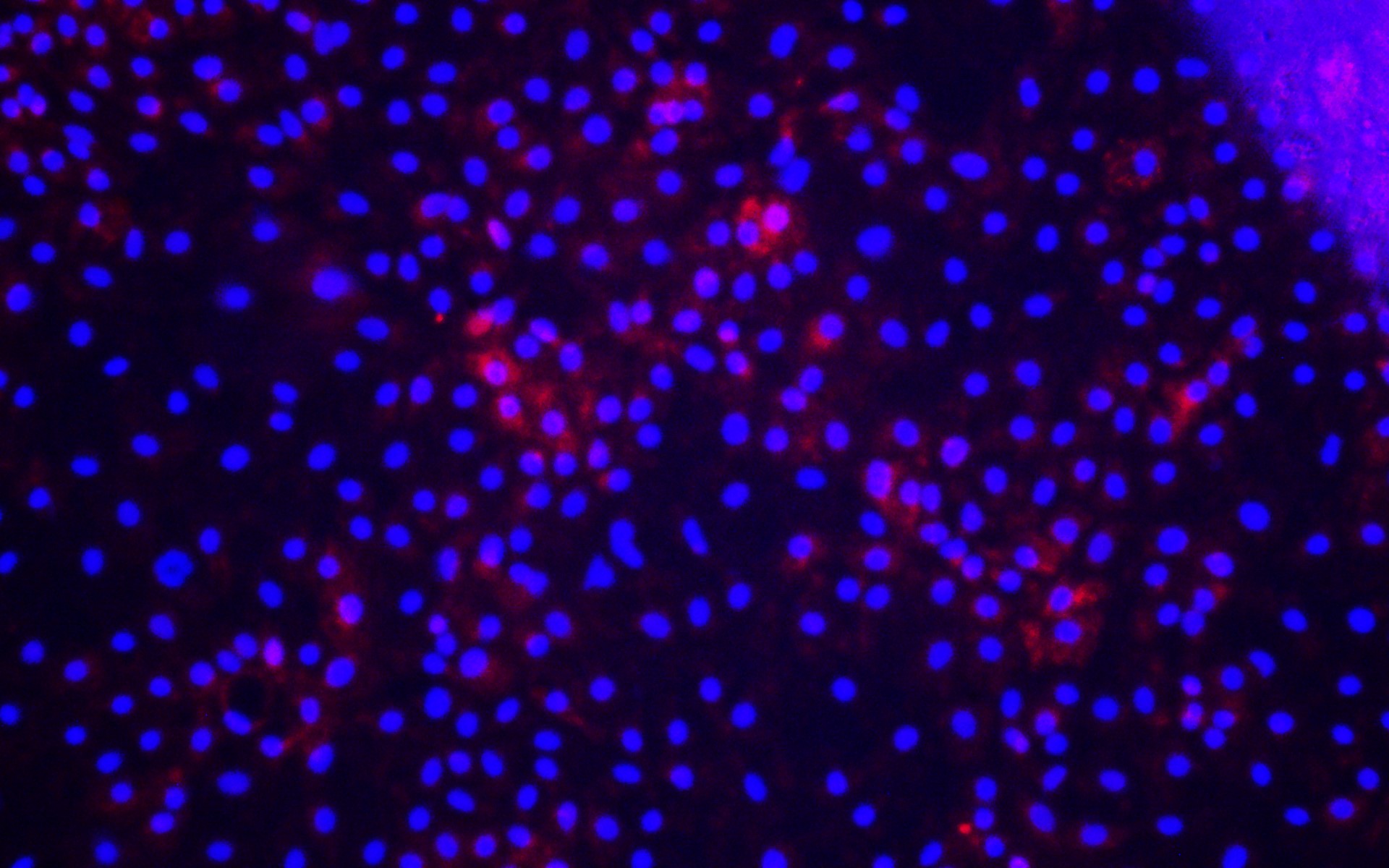

Supplement: Supplementary file 6 — Source data Fig. 4 [file 44319_2024_236_MOESM6_ESM.zip › Fig 4A/AFP/Hepatic specification/Merge_AFP(red)+nuclei.jpeg]

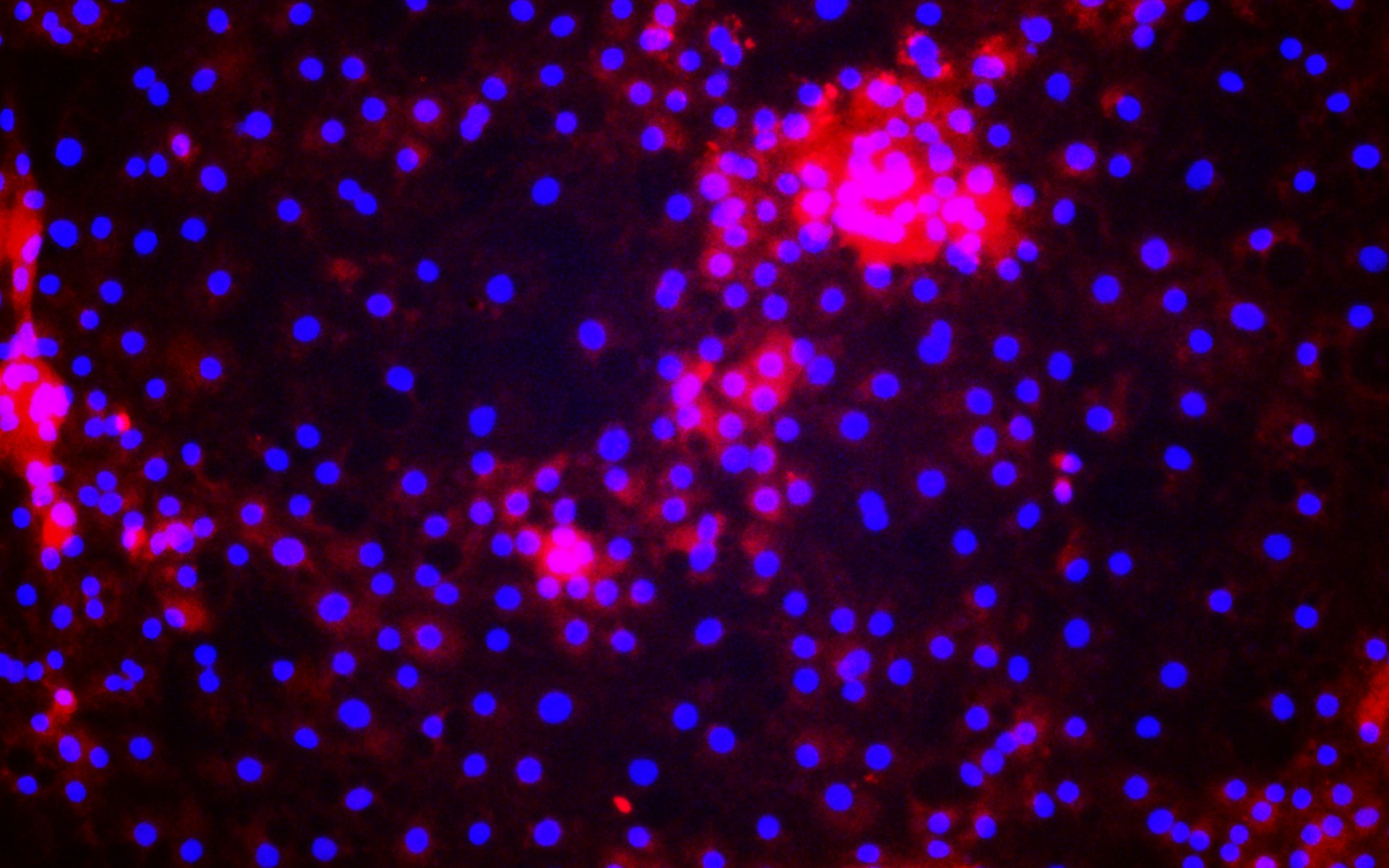

Supplement: Supplementary file 6 — Source data Fig. 4 [file 44319_2024_236_MOESM6_ESM.zip › Fig 4A/AFP/Hepatocyte like cell/Merge_AFP (red)+nuclei.jpeg]

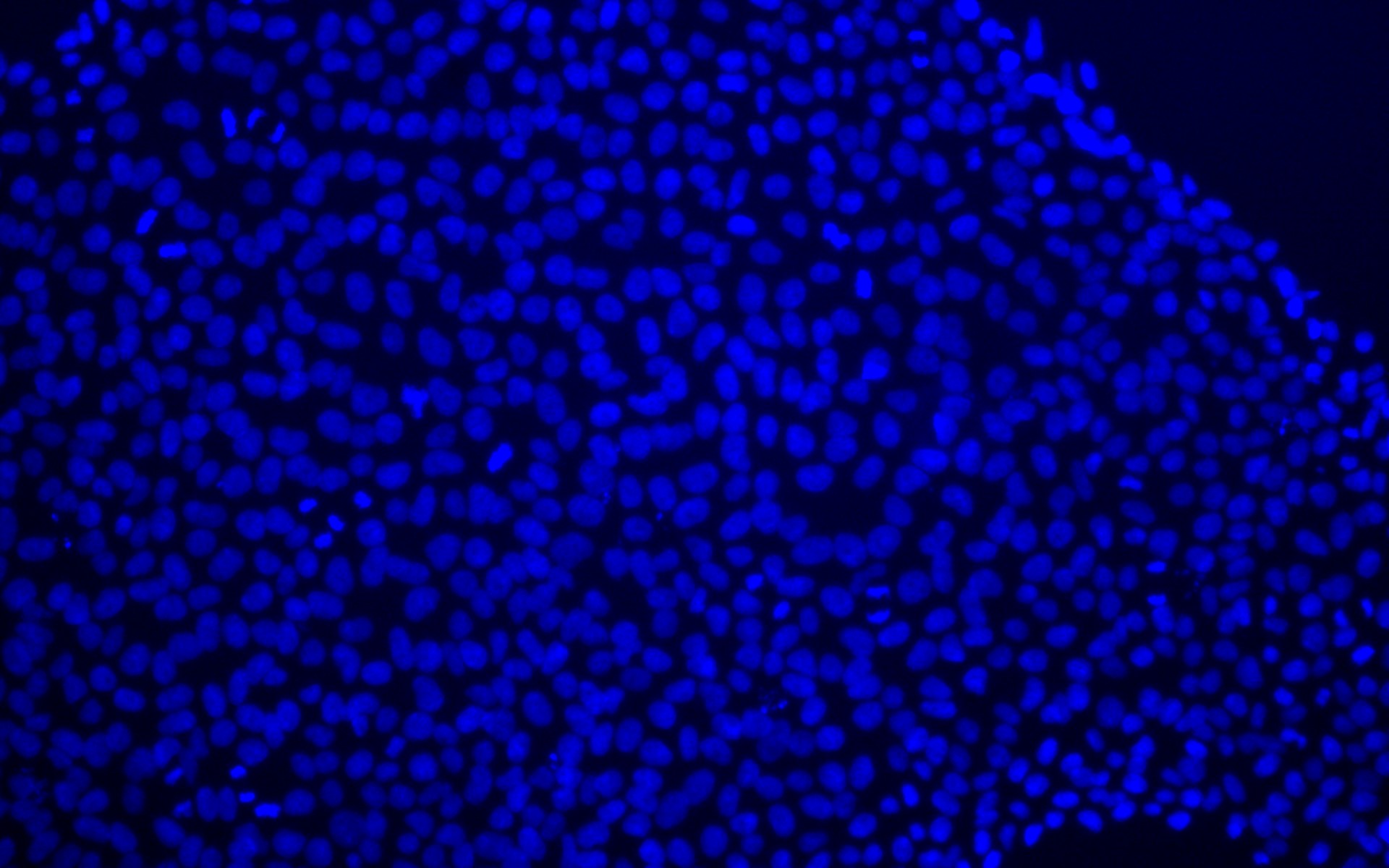

Supplement: Supplementary file 6 — Source data Fig. 4 [file 44319_2024_236_MOESM6_ESM.zip › Fig 4A/AFP/hESC/merge_AFP (red)+nuclei (blue).jpeg]

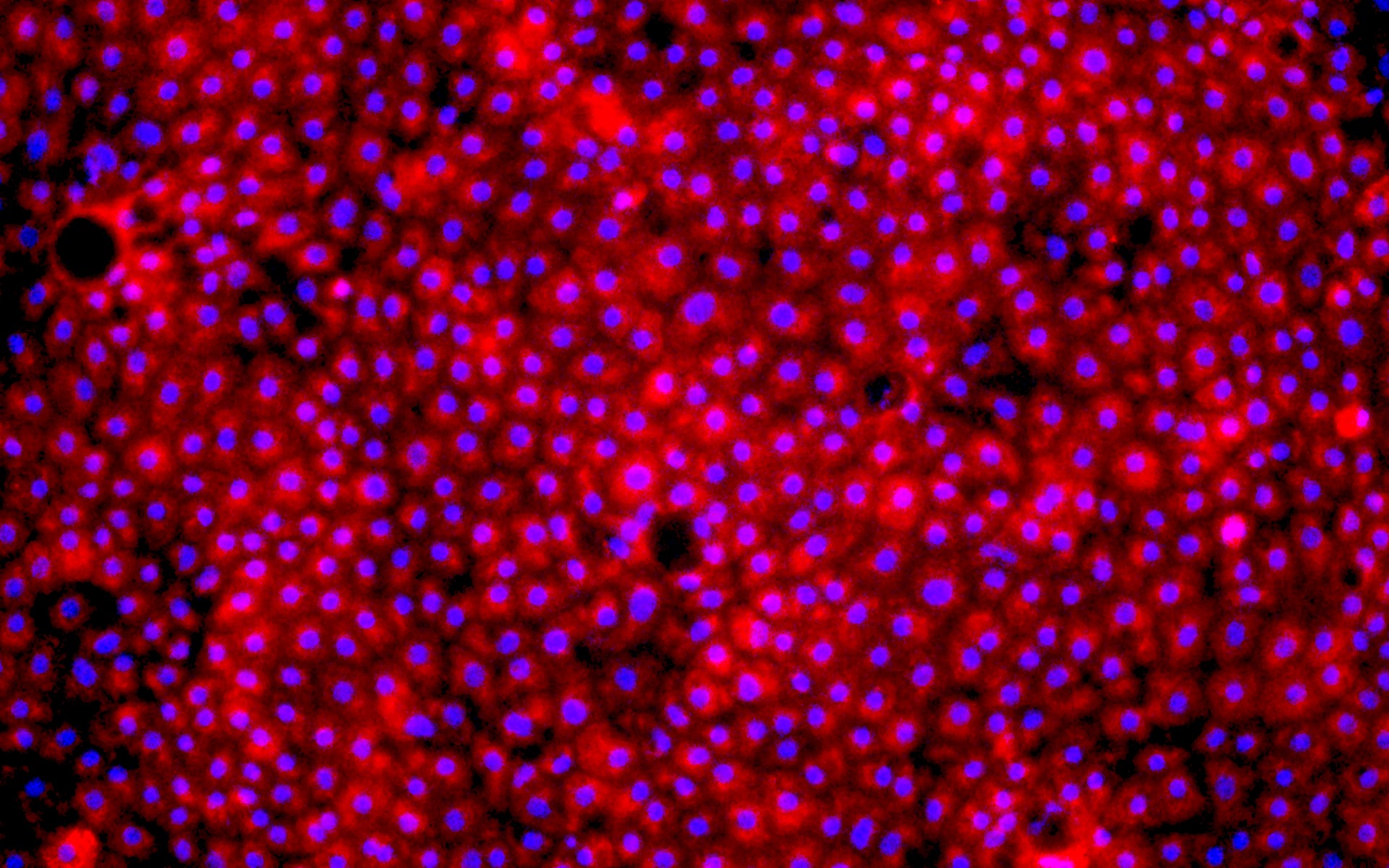

Supplement: Supplementary file 6 — Source data Fig. 4 [file 44319_2024_236_MOESM6_ESM.zip › Fig 4A/AFP/Immature hepatocyte/Merge_AFP (red) +nuclei.jpeg]

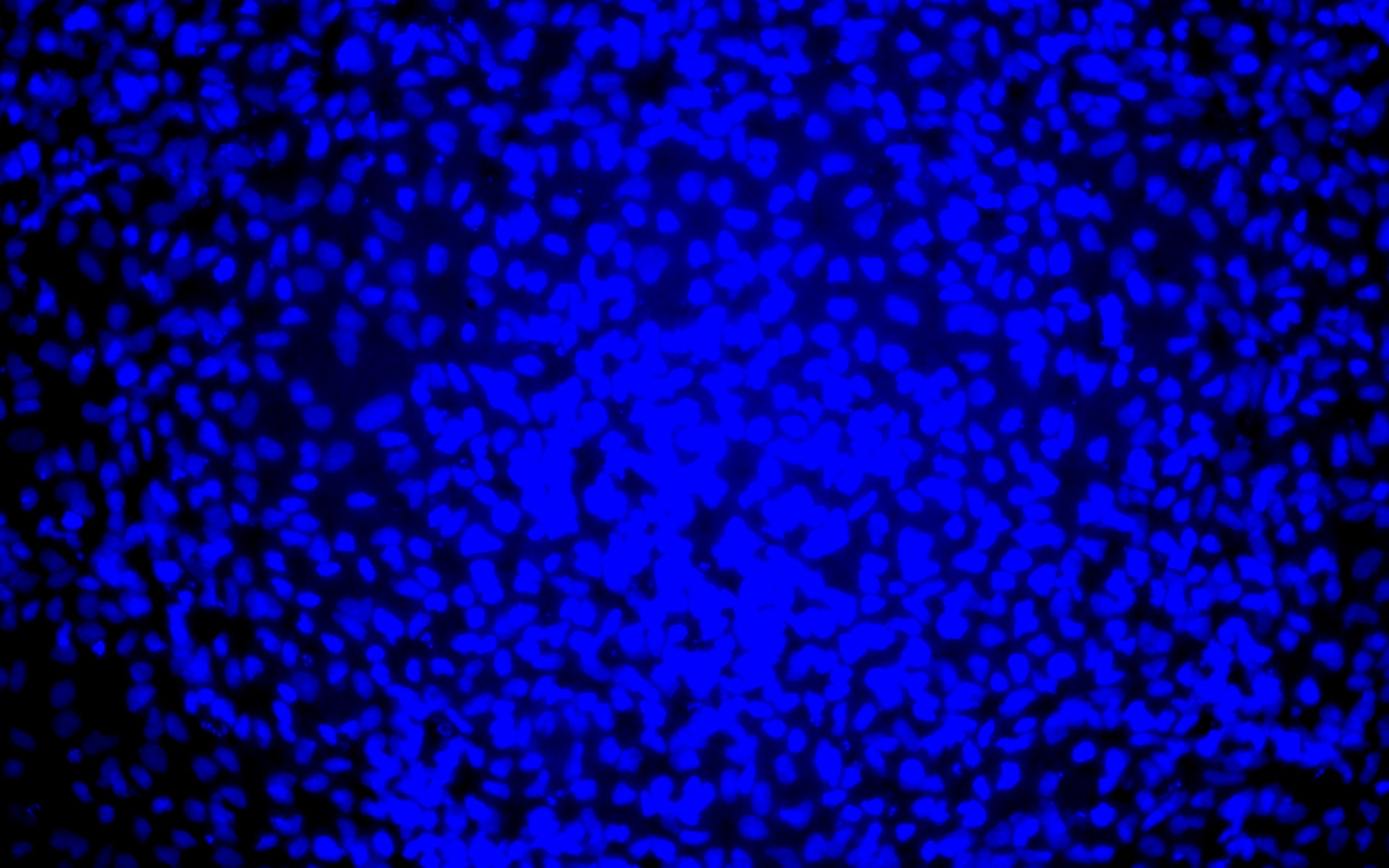

Supplement: Supplementary file 6 — Source data Fig. 4 [file 44319_2024_236_MOESM6_ESM.zip › Fig 4A/ALB/definite endoderm/Merge_ALB (red)+nuclei.jpeg]

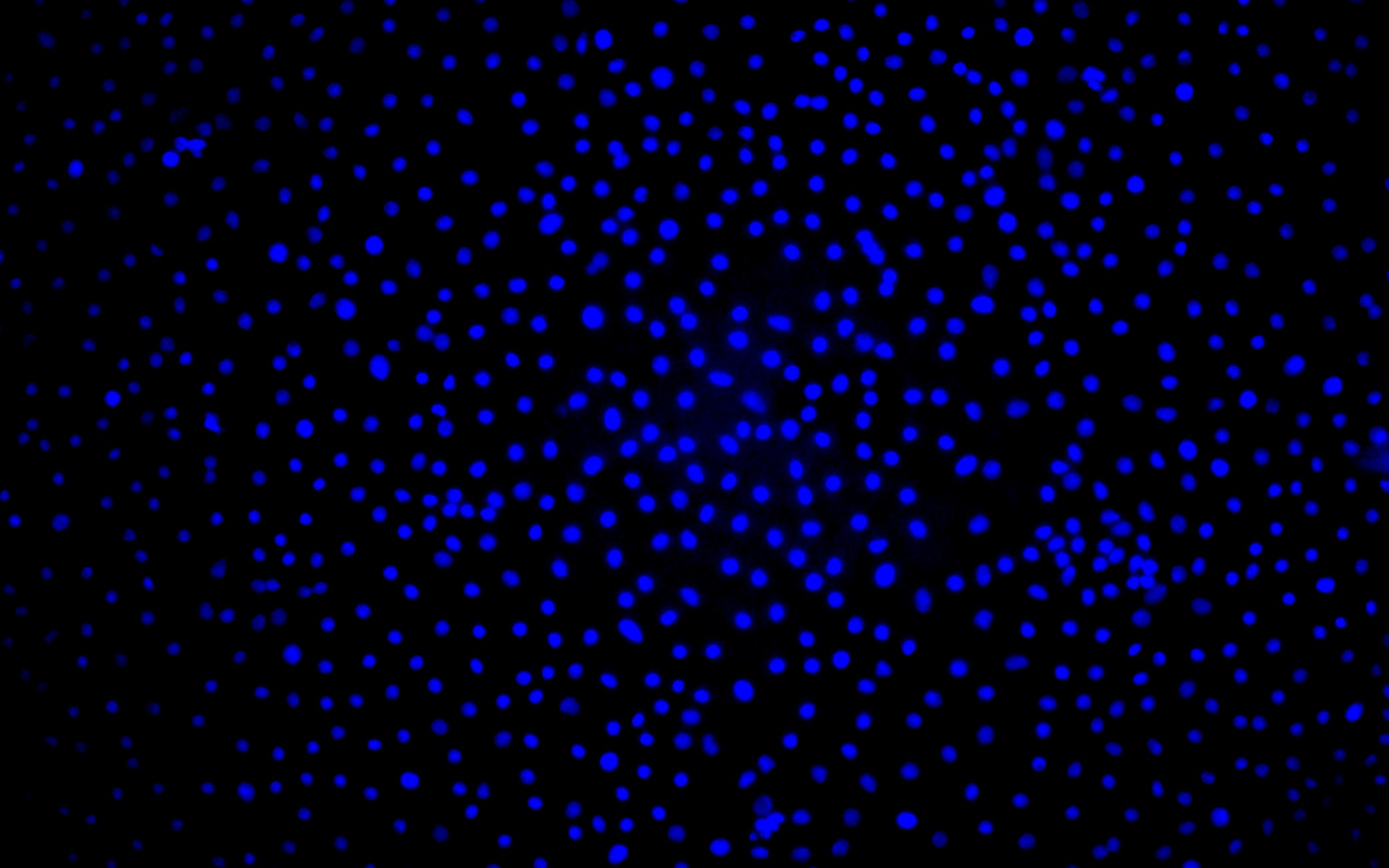

Supplement: Supplementary file 6 — Source data Fig. 4 [file 44319_2024_236_MOESM6_ESM.zip › Fig 4A/ALB/Hepatic specification/Merge_ALB (red)+nuclei.jpeg]

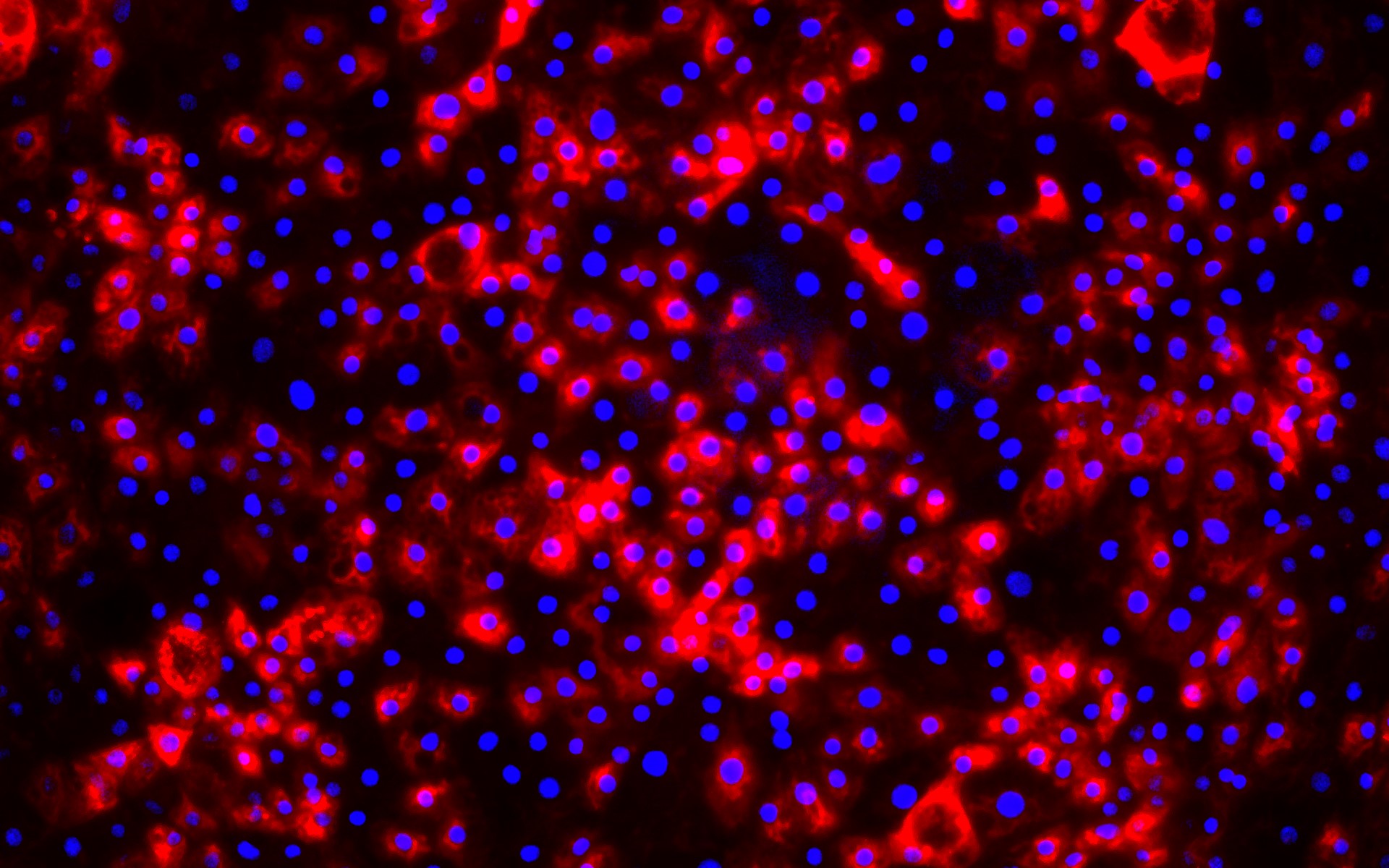

Supplement: Supplementary file 6 — Source data Fig. 4 [file 44319_2024_236_MOESM6_ESM.zip › Fig 4A/ALB/Hepatocyte like cell/Merge_ALB (red)+nuclei.jpeg]

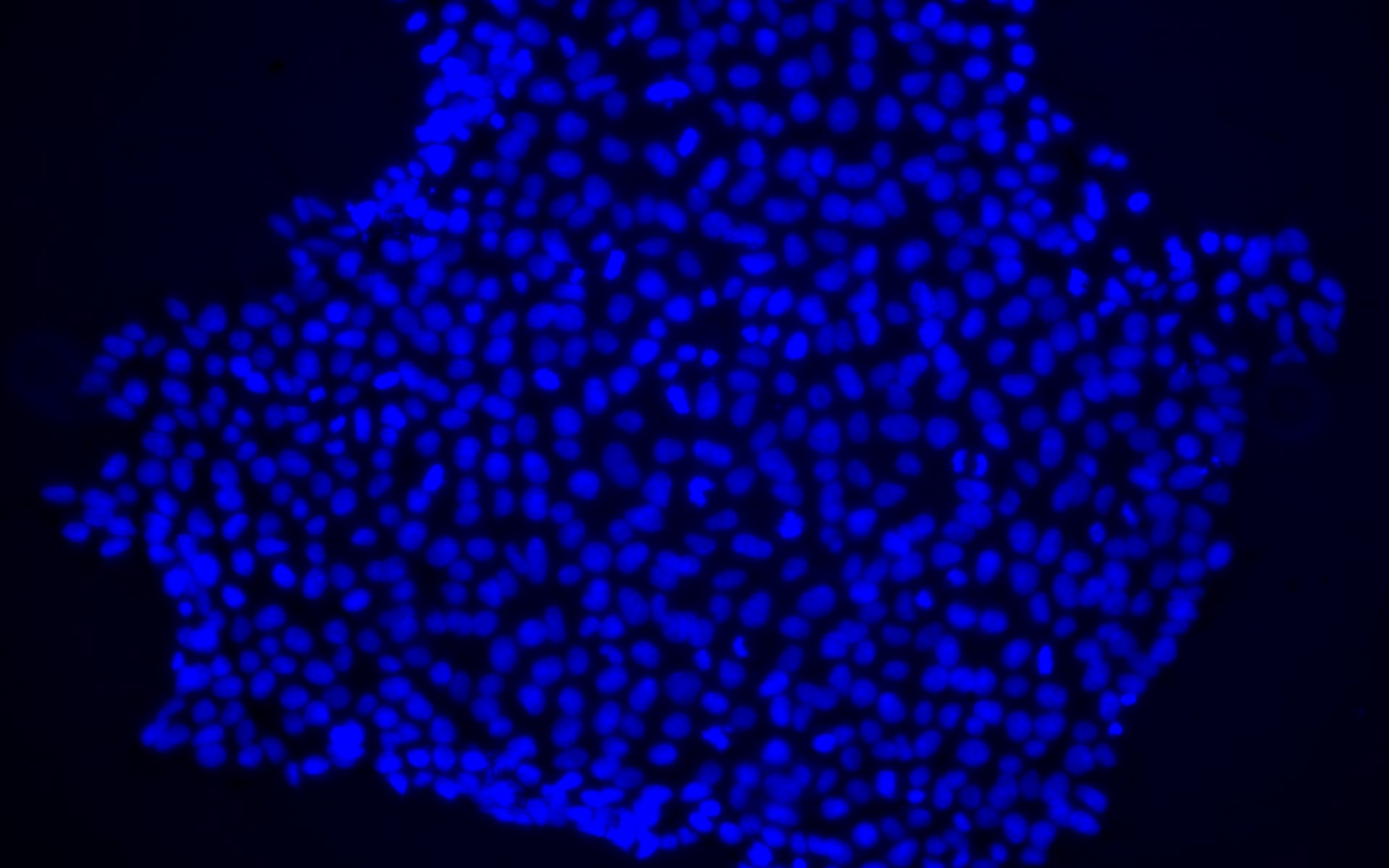

Supplement: Supplementary file 6 — Source data Fig. 4 [file 44319_2024_236_MOESM6_ESM.zip › Fig 4A/ALB/hESC/Merge_ALB (red)+nuclei.jpeg]

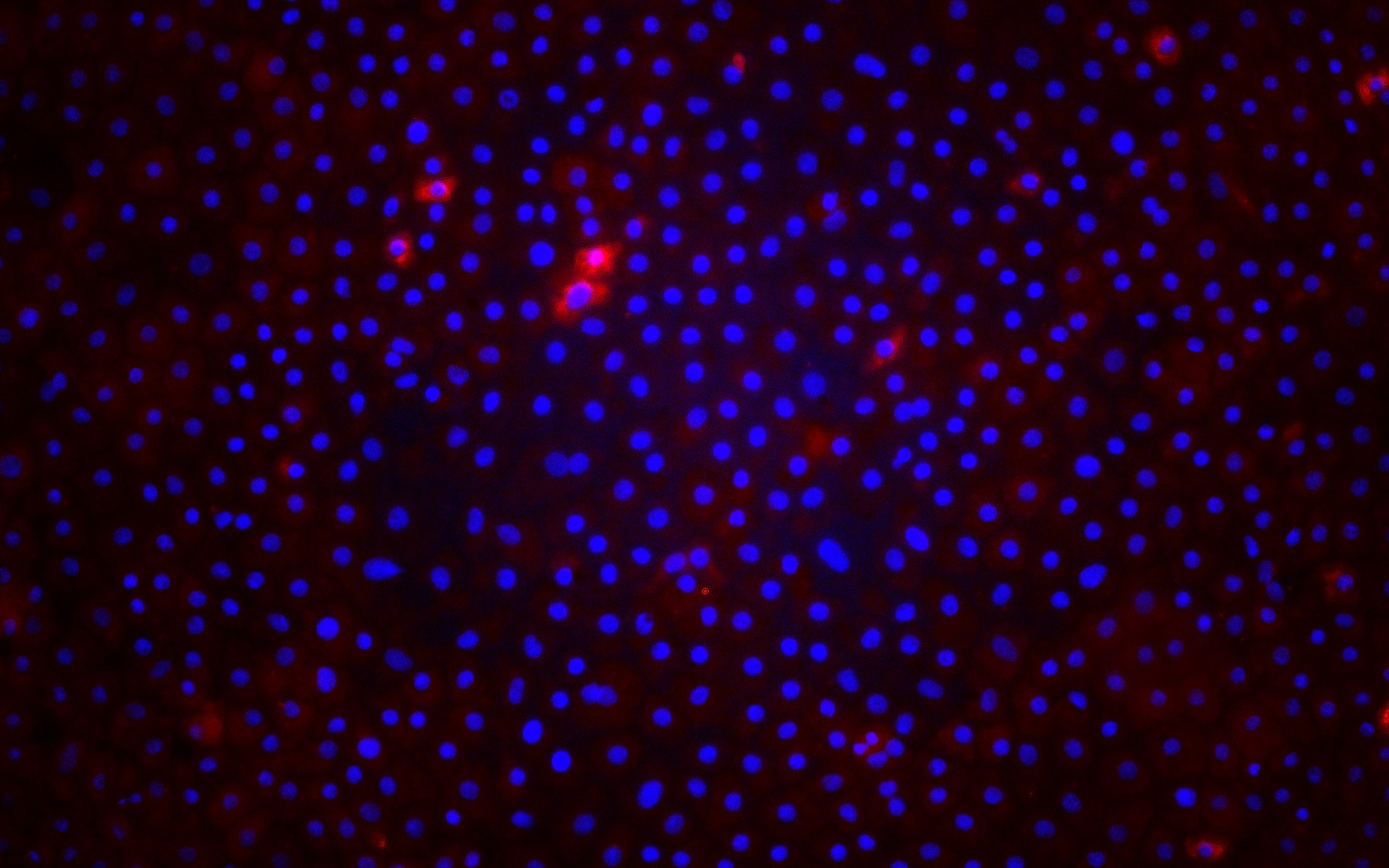

Supplement: Supplementary file 6 — Source data Fig. 4 [file 44319_2024_236_MOESM6_ESM.zip › Fig 4A/ALB/Immature hepatocyte/Merge_ALB (red)+nuclei.jpeg]

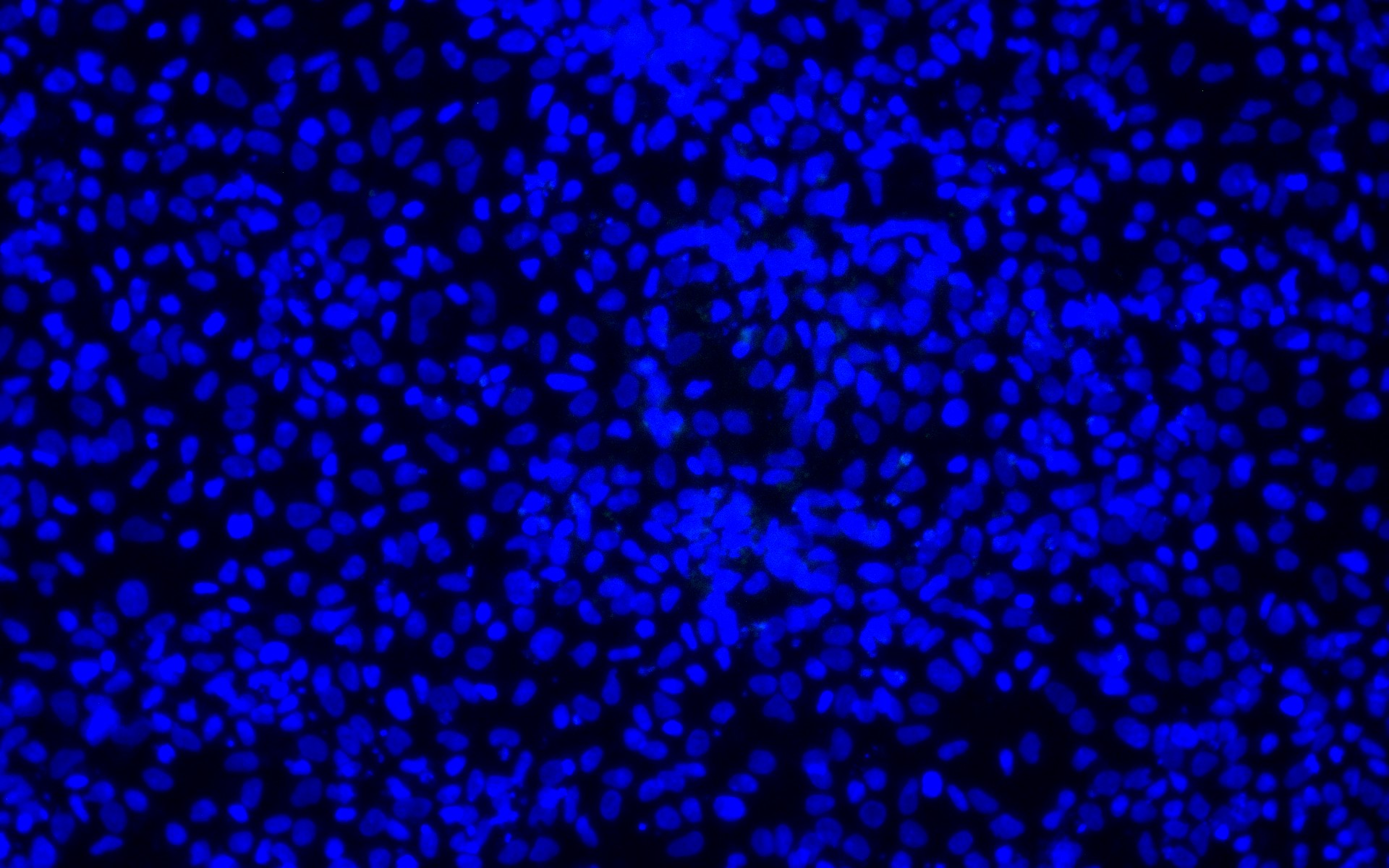

Supplement: Supplementary file 6 — Source data Fig. 4 [file 44319_2024_236_MOESM6_ESM.zip › Fig 4A/NTCP/definite endoderm/Merge_NTCP (green)+nuclei.jpeg]

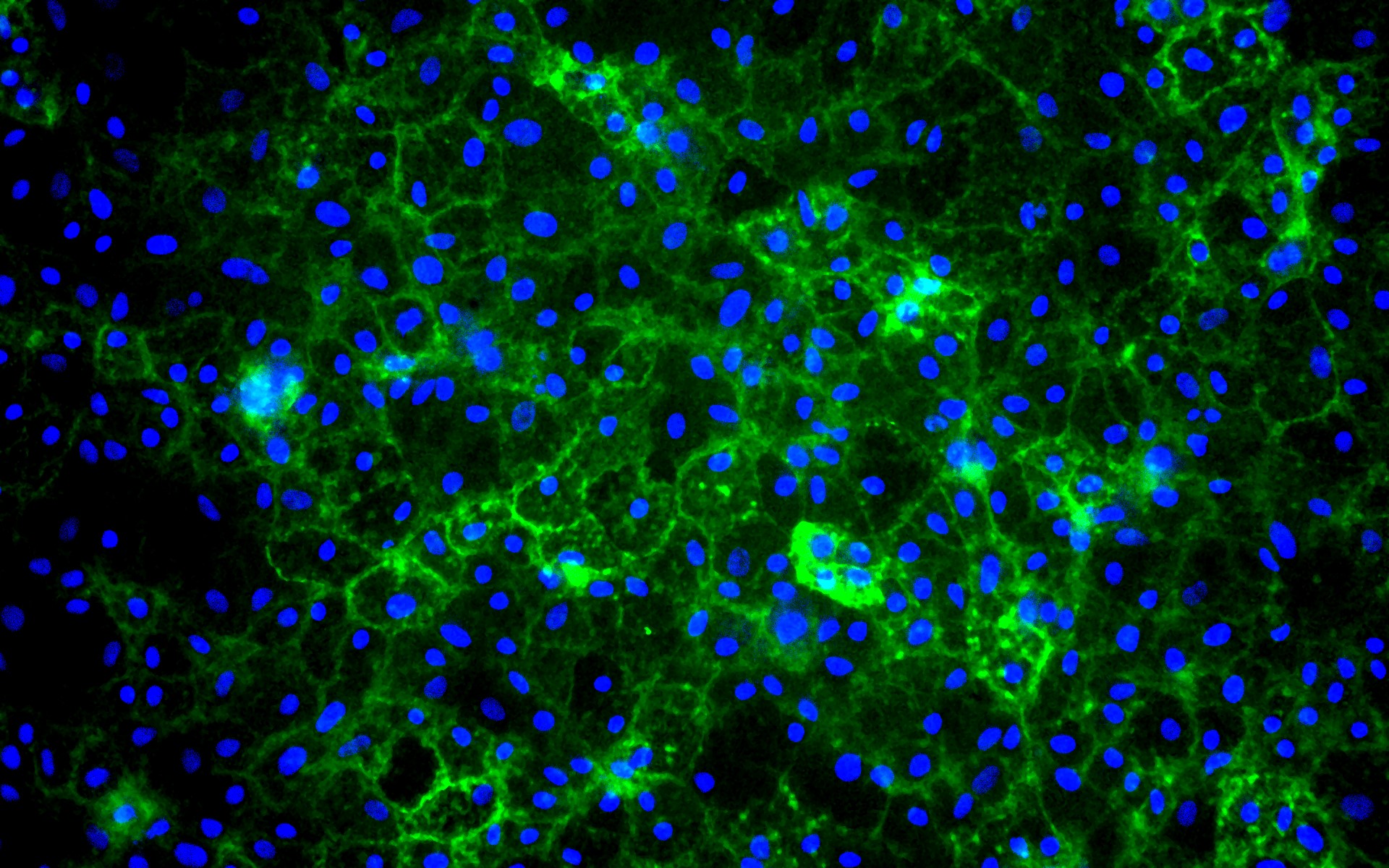

Supplement: Supplementary file 6 — Source data Fig. 4 [file 44319_2024_236_MOESM6_ESM.zip › Fig 4A/NTCP/Hepatic specification/Merge_NTCP(green)+nuclei.jpeg]

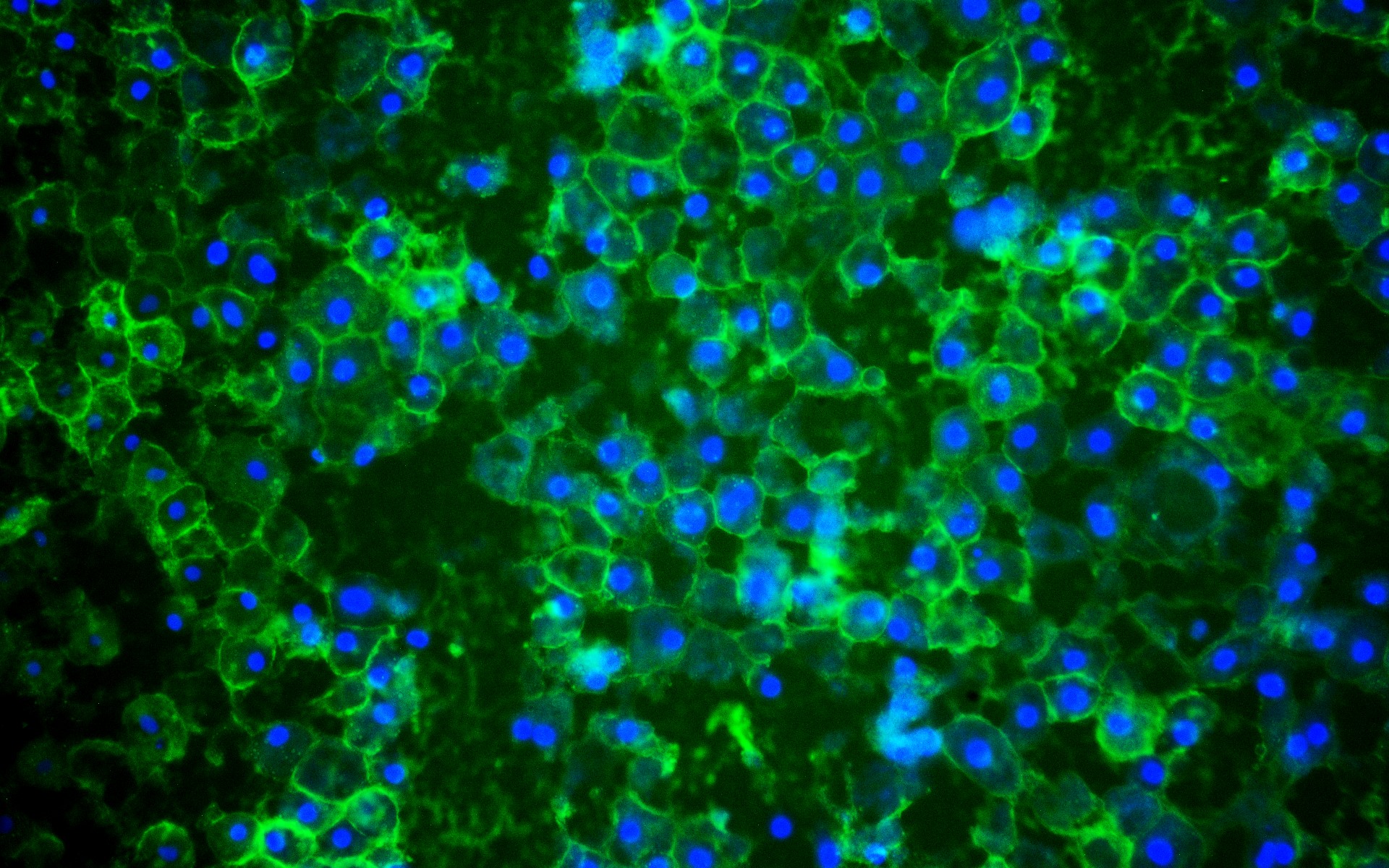

Supplement: Supplementary file 6 — Source data Fig. 4 [file 44319_2024_236_MOESM6_ESM.zip › Fig 4A/NTCP/Hepatocyte like cell/Merge_NTCP+nuclei.jpeg]

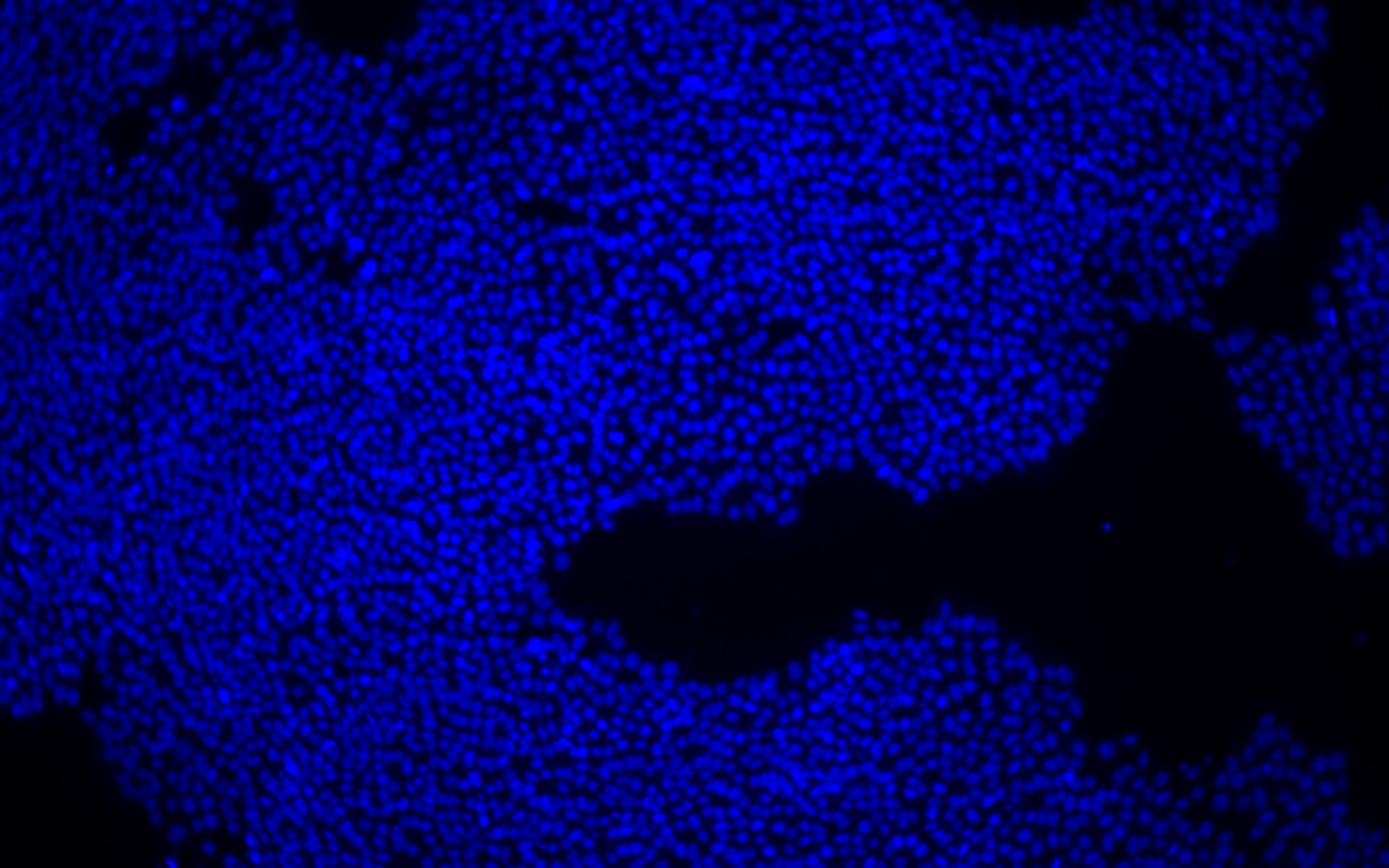

Supplement: Supplementary file 6 — Source data Fig. 4 [file 44319_2024_236_MOESM6_ESM.zip › Fig 4A/NTCP/hESC/Merge_NTCP(green)+nuclei.jpeg]

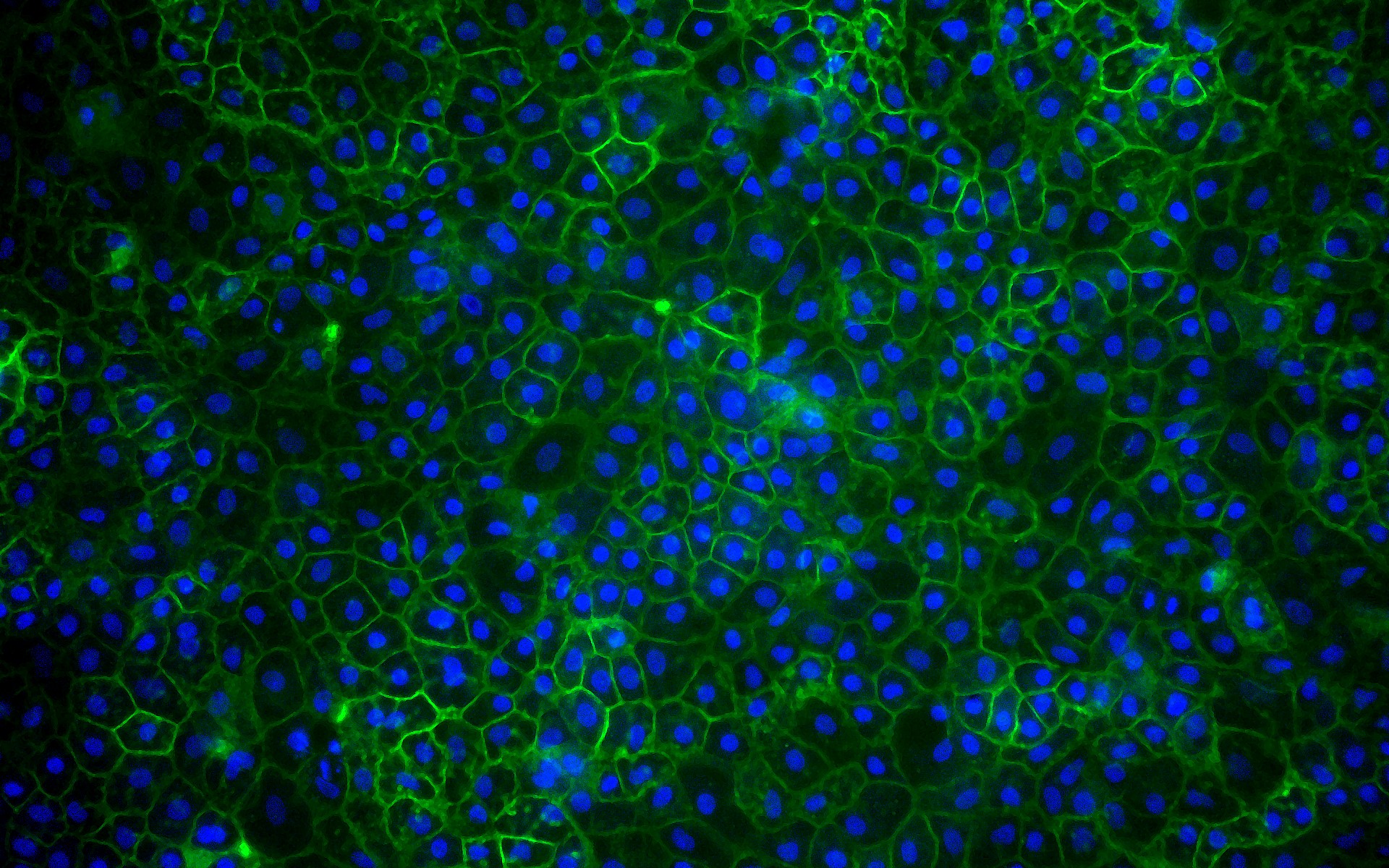

Supplement: Supplementary file 6 — Source data Fig. 4 [file 44319_2024_236_MOESM6_ESM.zip › Fig 4A/NTCP/Immature hepatocyte/Merge_NTCP(green)+nuclei.jpeg]

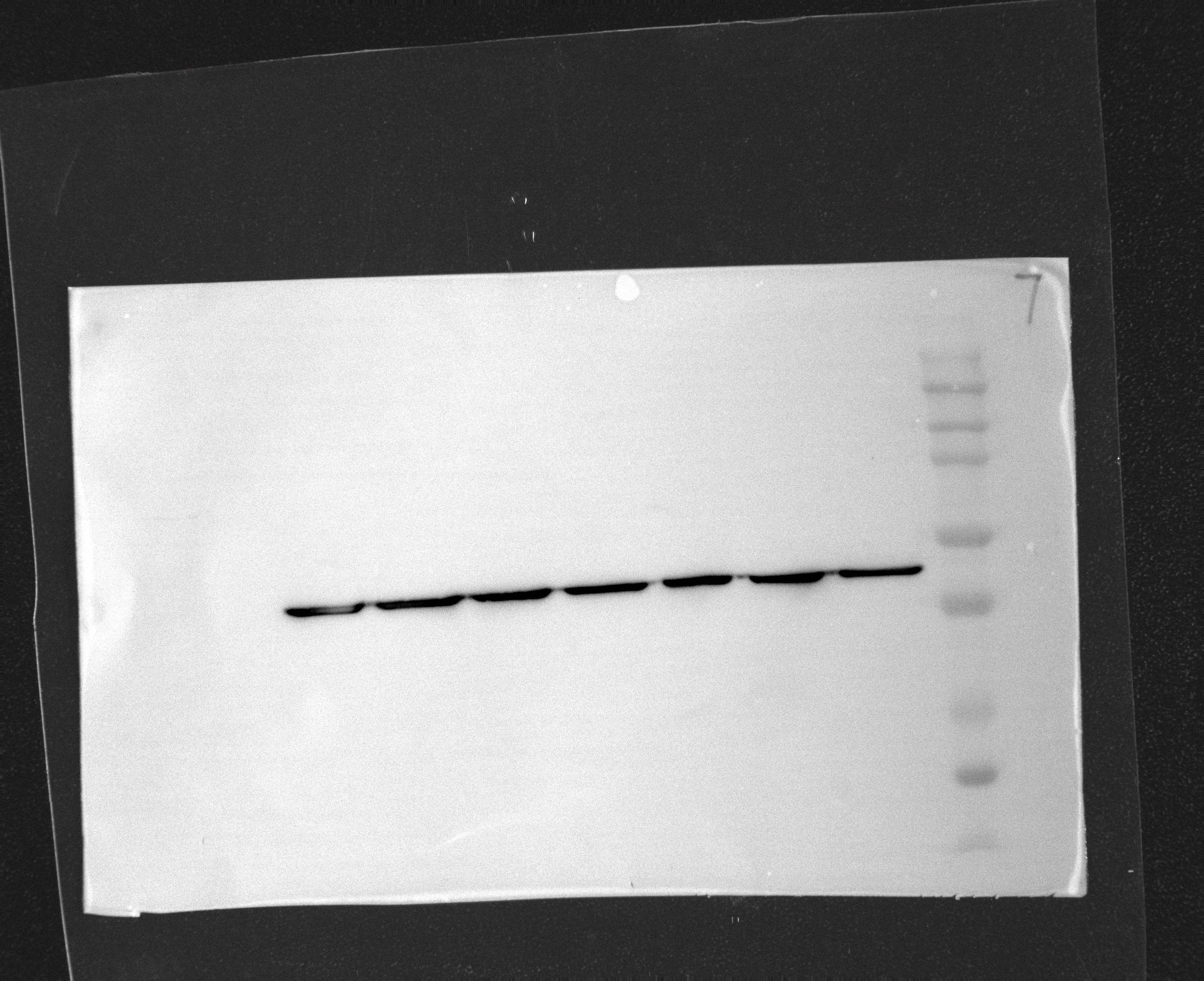

Supplement: Supplementary file 7 — Source data Fig. 5 [file 44319_2024_236_MOESM7_ESM.zip › FIg 5F/bactin/bactin+ladder_image.tif]

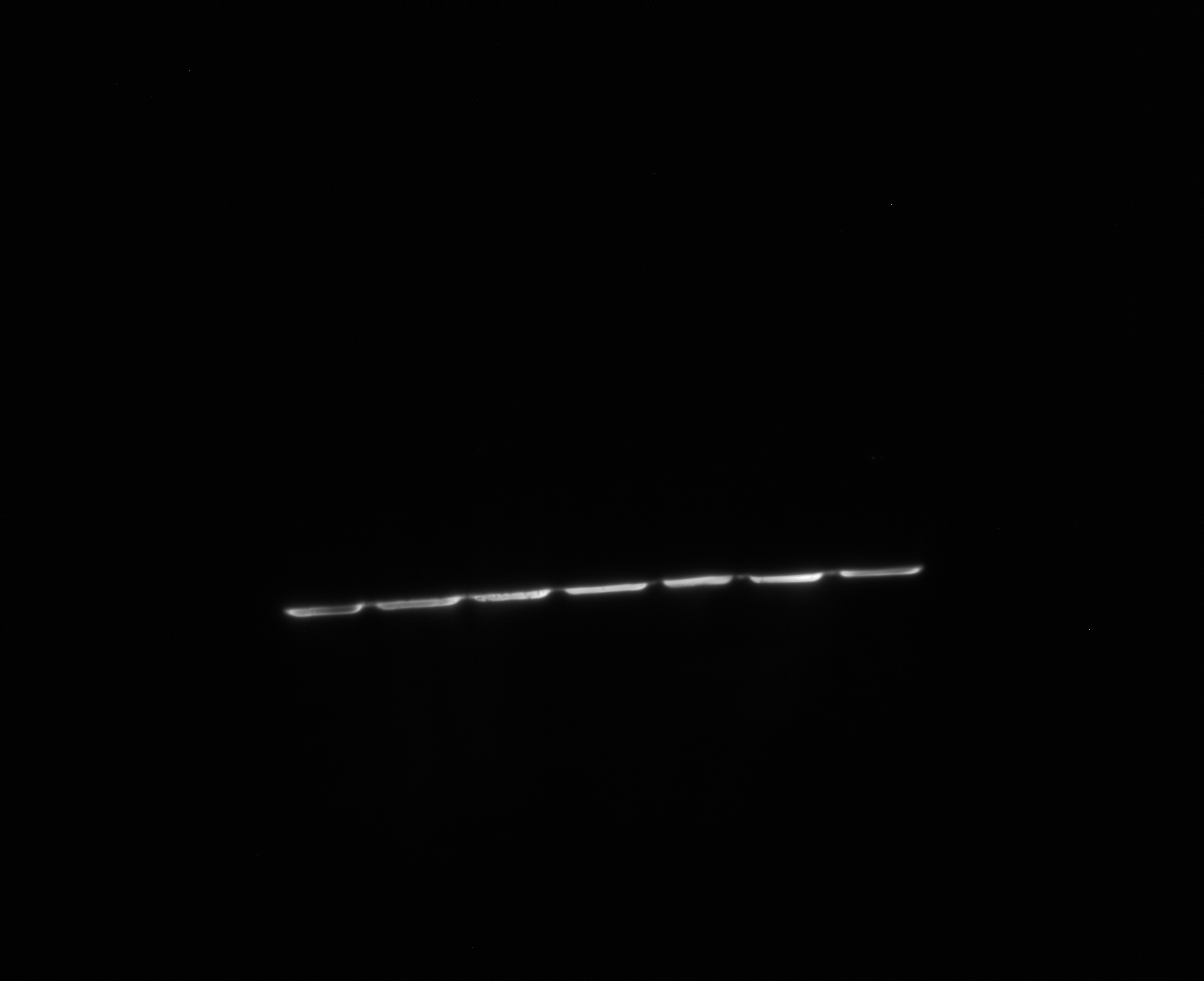

Supplement: Supplementary file 7 — Source data Fig. 5 [file 44319_2024_236_MOESM7_ESM.zip › FIg 5F/bactin/bactin_raw data.tif]

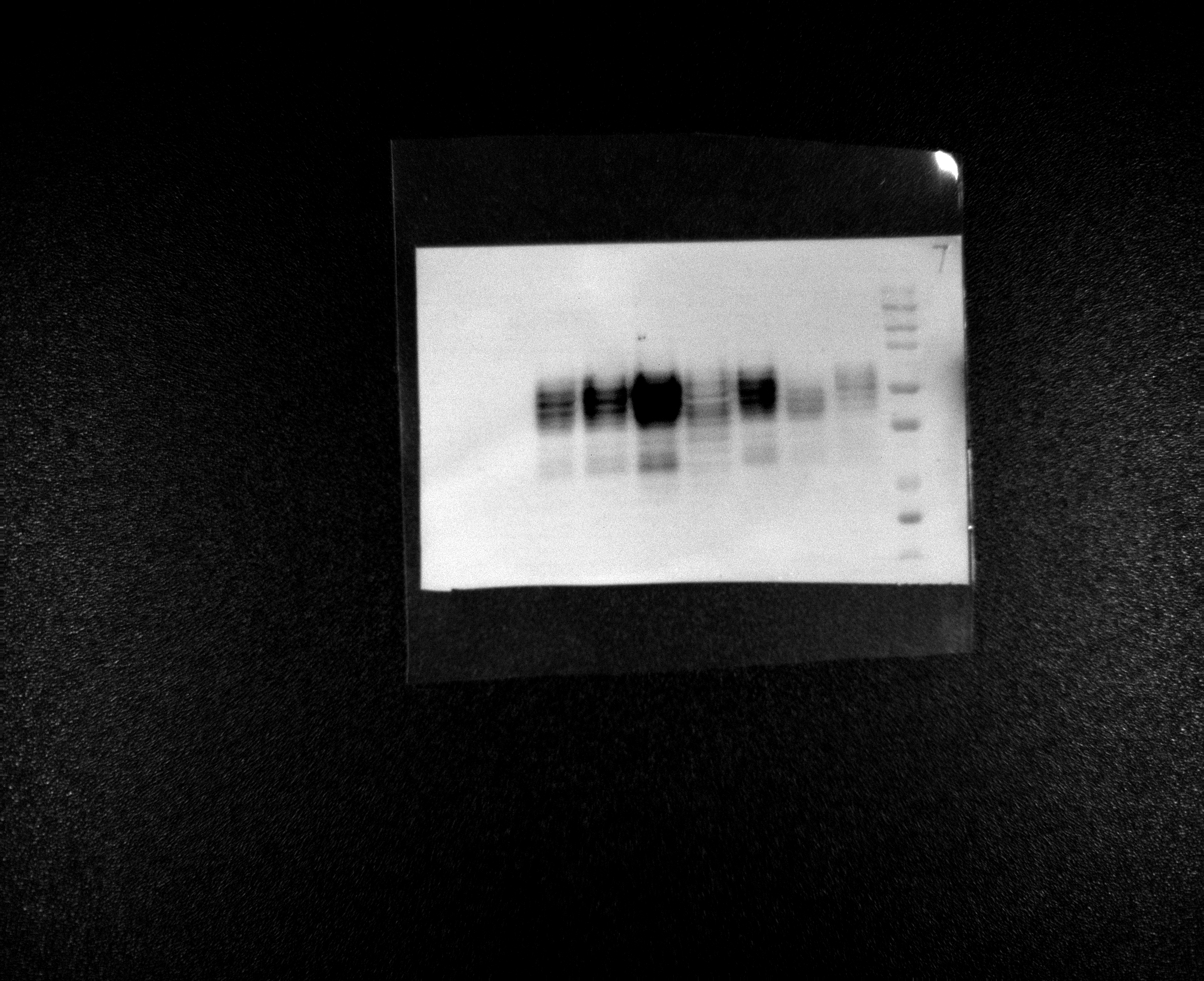

Supplement: Supplementary file 7 — Source data Fig. 5 [file 44319_2024_236_MOESM7_ESM.zip › FIg 5F/CD63/CD63+ladder_image.tif]

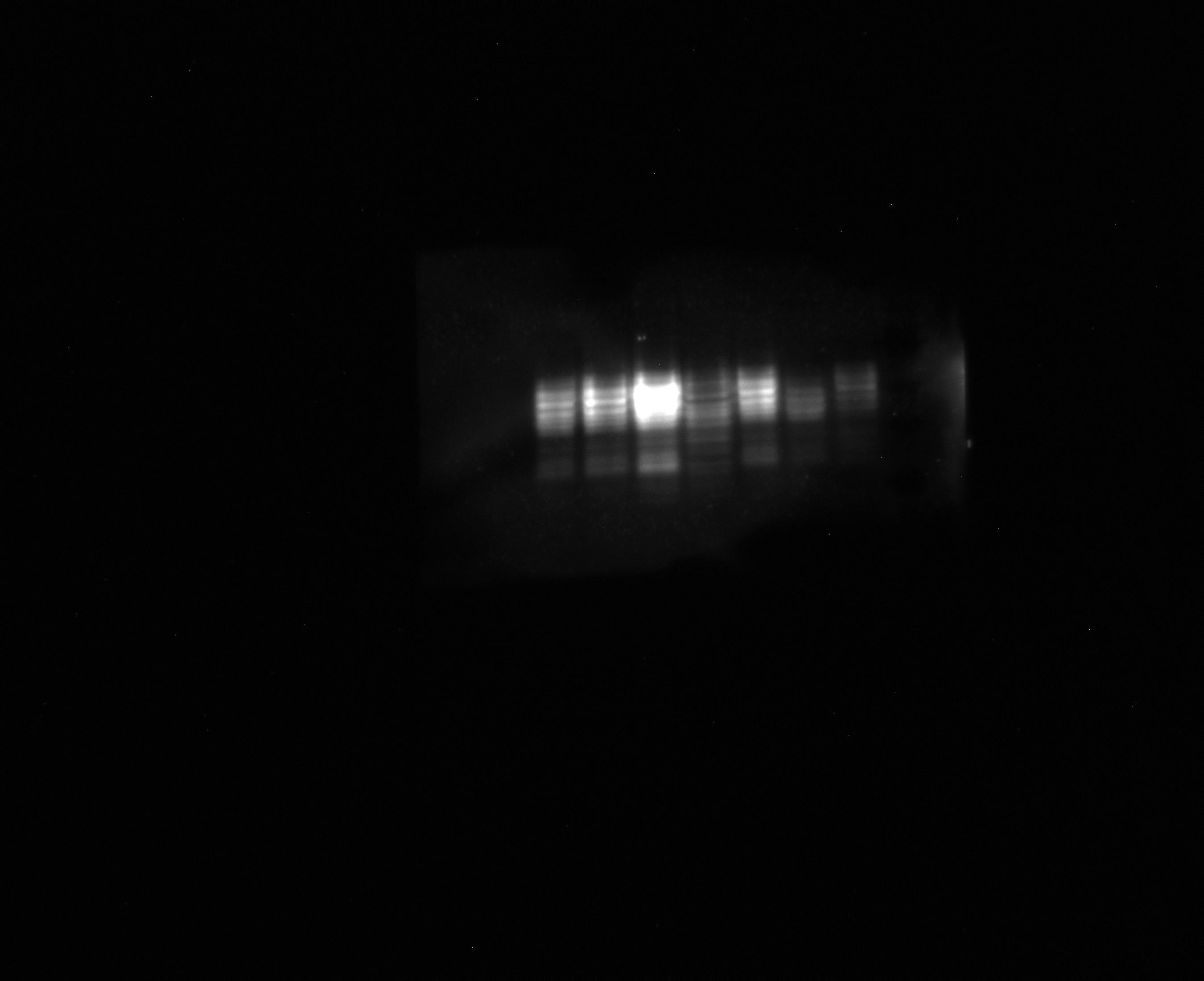

Supplement: Supplementary file 7 — Source data Fig. 5 [file 44319_2024_236_MOESM7_ESM.zip › FIg 5F/CD63/CD63_raw data.tif]

## Slide 1
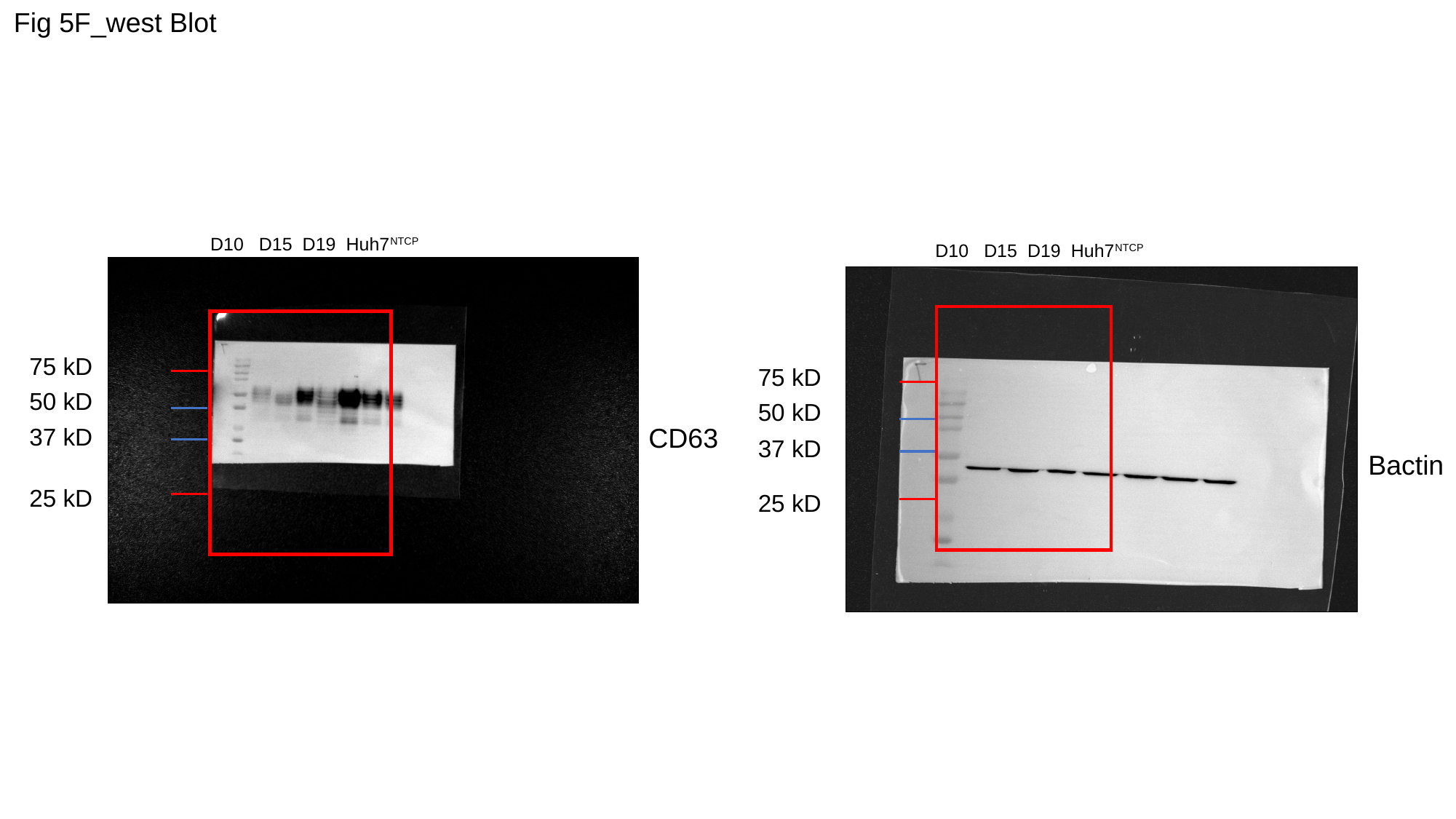

Fig 5F_west Blot
D10 D15 D19 Huh7NTCP
D10 D15 D19 Huh7NTCP
75 kD
75 kD
50 kD
50 kD
CD63
37 kD
37 kD
Bactin
25 kD
25 kD

Supplement: Supplementary file 7 — Source data Fig. 5 [file 44319_2024_236_MOESM7_ESM.zip › FIg 5F/Fig 5F.pptx]

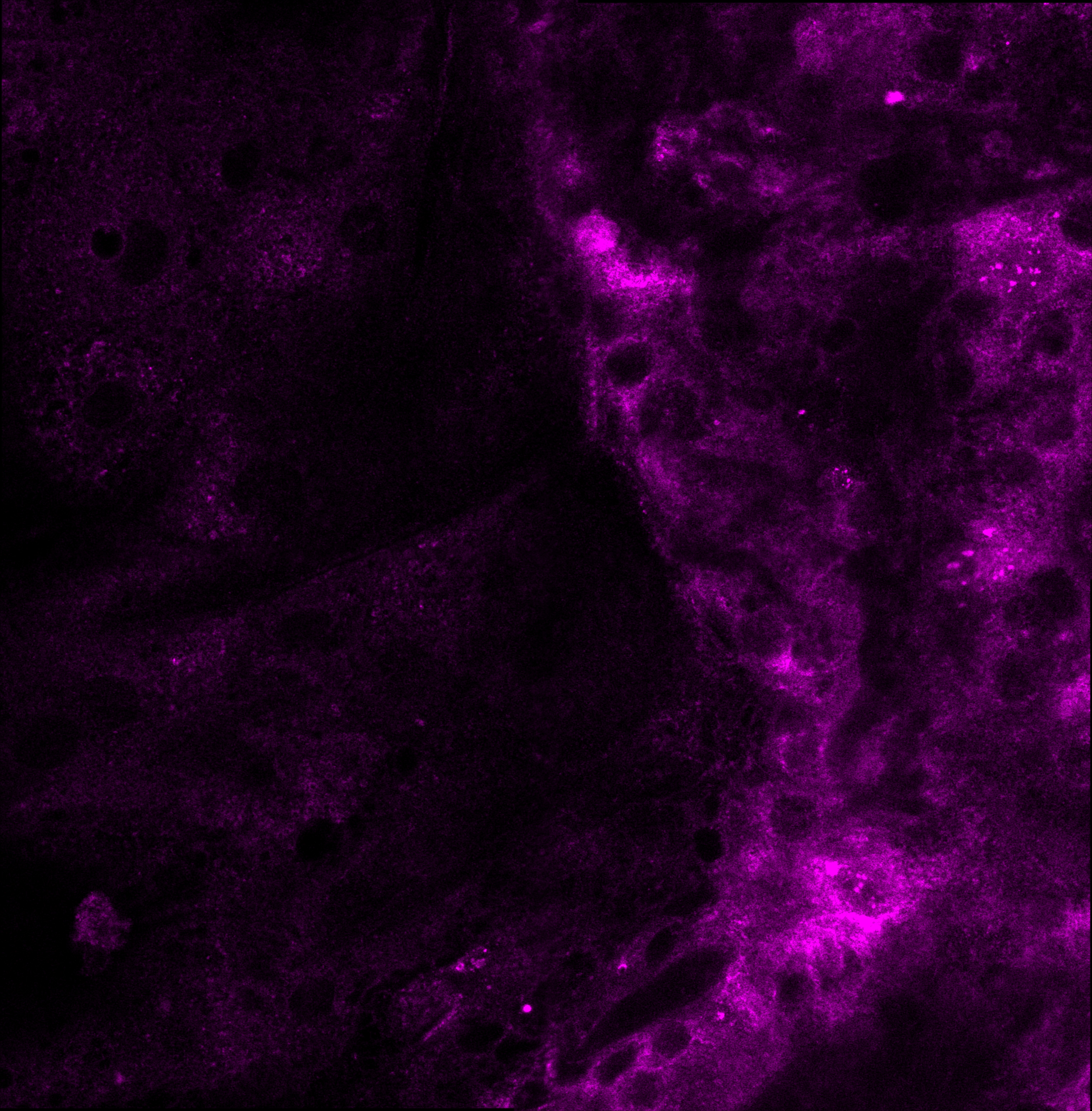

Supplement: Supplementary file 7 — Source data Fig. 5 [file 44319_2024_236_MOESM7_ESM.zip › Fig 5G/CD63 (magenta).jpeg]

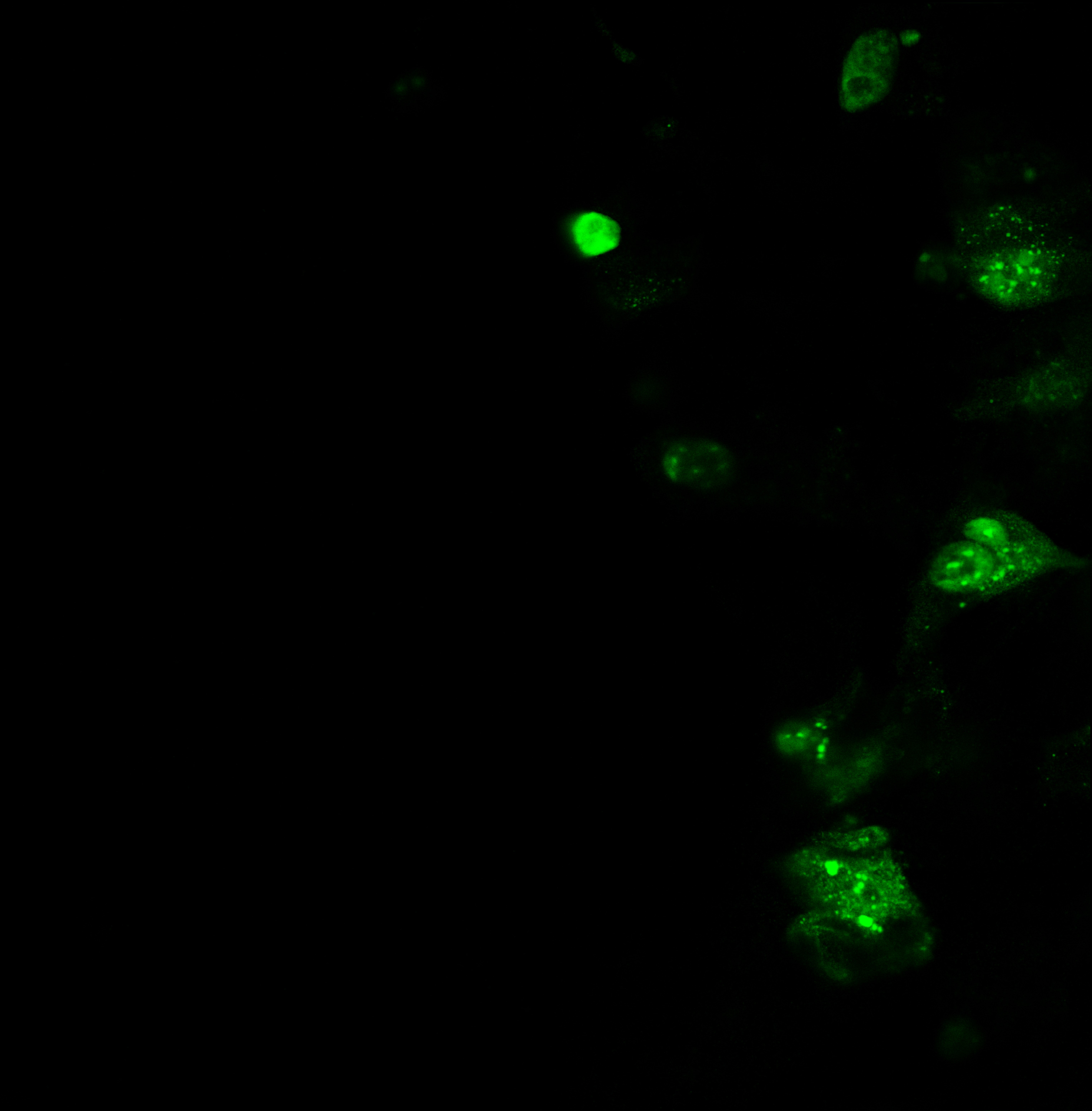

Supplement: Supplementary file 7 — Source data Fig. 5 [file 44319_2024_236_MOESM7_ESM.zip › Fig 5G/HDAg (green).jpeg]

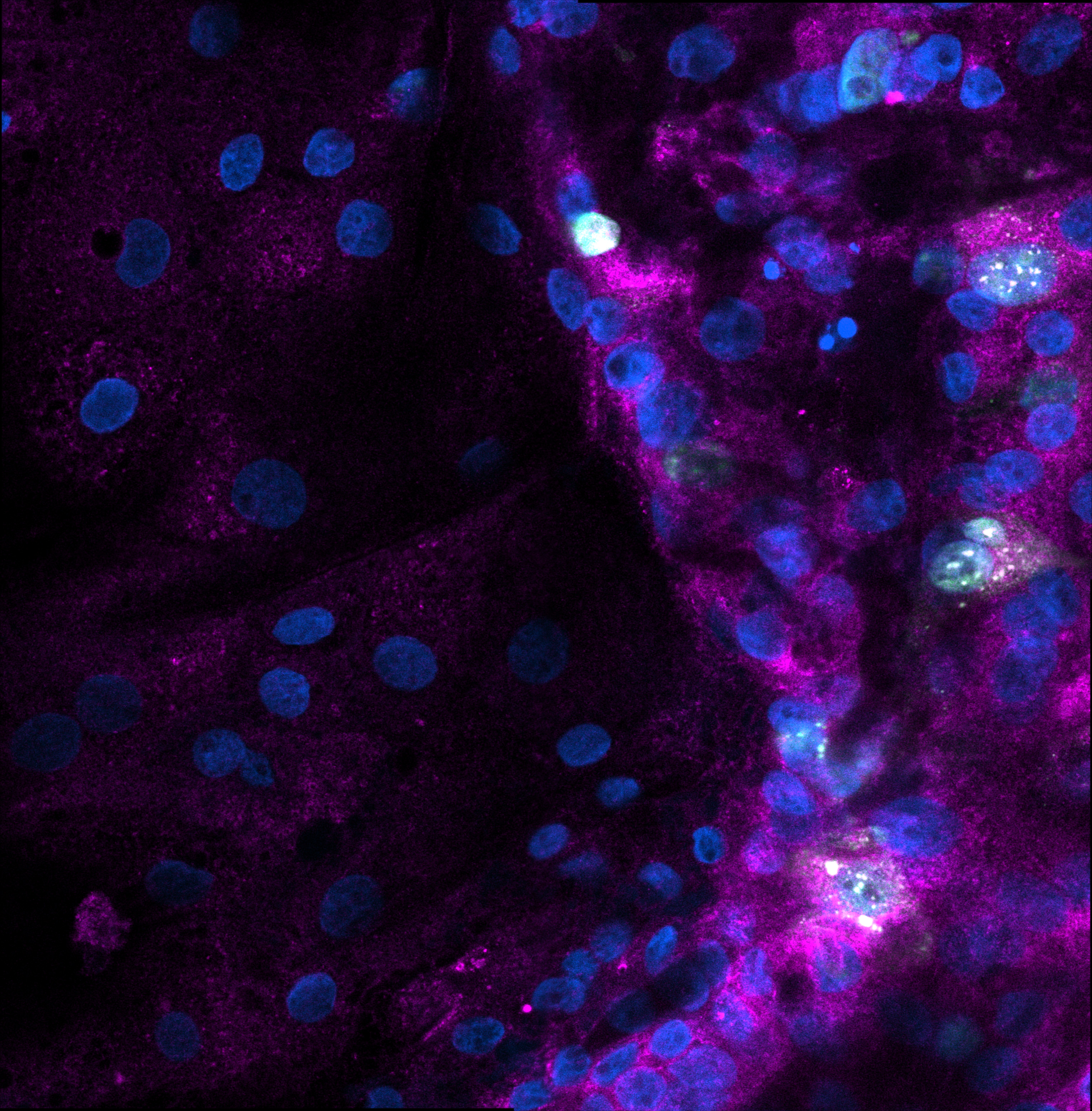

Supplement: Supplementary file 7 — Source data Fig. 5 [file 44319_2024_236_MOESM7_ESM.zip › Fig 5G/Merge-HDAg+CD63+nuclei (1).jpeg]

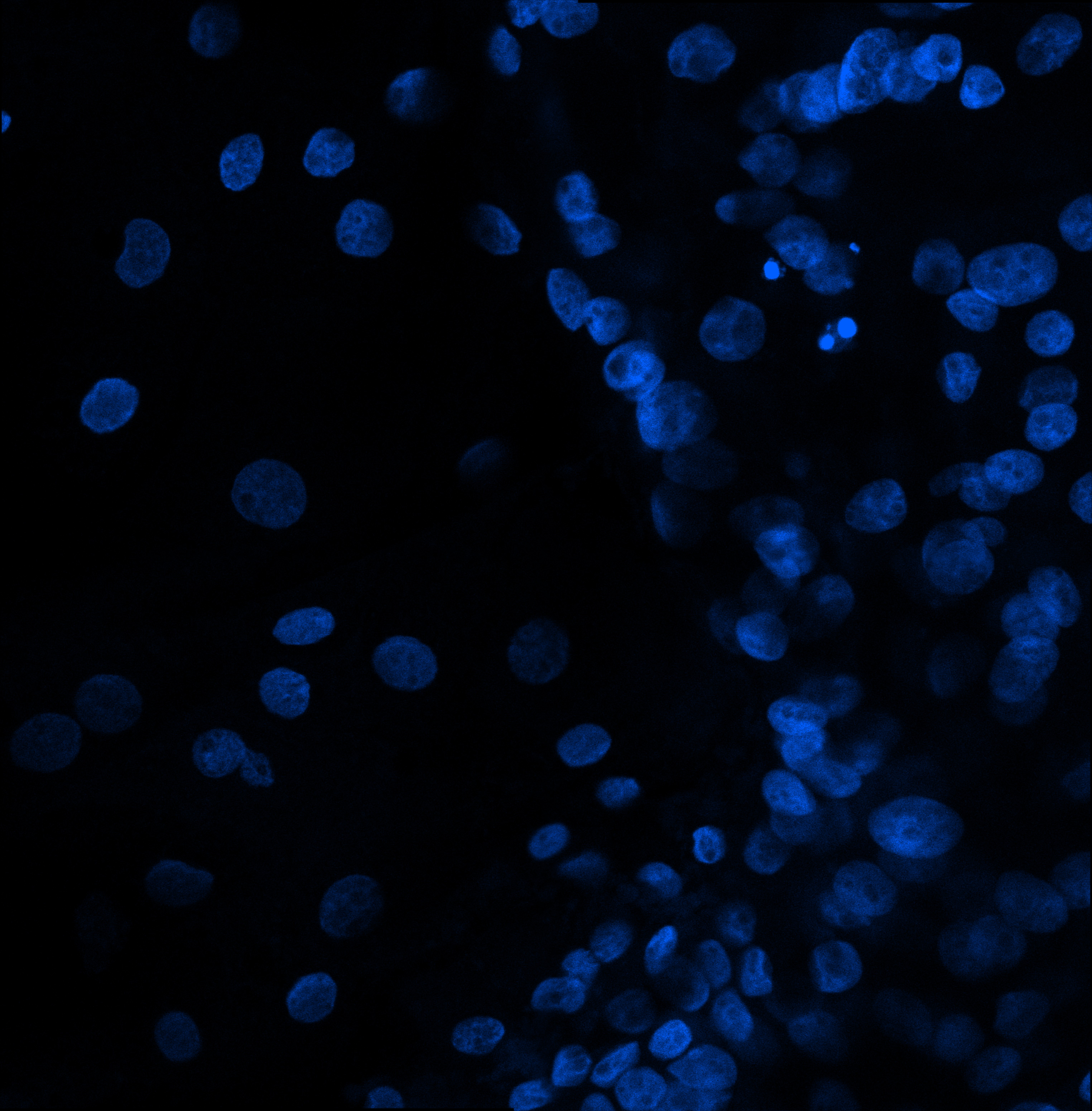

Supplement: Supplementary file 7 — Source data Fig. 5 [file 44319_2024_236_MOESM7_ESM.zip › Fig 5G/nuclei (blue).jpeg]
