# Supplementary material for: An hepatitis B and D virus infection model using human pluripotent stem cell-derived hepatocytes
Source: EMBO Rep. 2024 Sep 4;25(10):17. doi: 10.1038/s44319-024-00236-0 (PMC11466959; doi:10.1038/s44319-024-00236-0)
Supplement: Supplementary file 8 — Expanded View Figures [file 44319_2024_236_MOESM8_ESM.pdf]

Expanded View Figures

Figure EV1. HLCs are susceptible for HDV infection.

(**A**, **B**) HLCs were infected with HDV at different MOIs (0.5, 1, 2.5, or 5 Int. Units/cell) and HDV antigen levels were analyzed (HDAg, red) 5 days p.i. Scale bar = 100 µm. HDAg-positive cells were counted using CellProfiler. $N = 6$ biological replicates from two independent HLC differentiations. (**C**) HLCs, differentiated HepaRG, and Huh7^{NTCP} cells were infected with HDV (MOI = 5 Int. Units/cell). HDAg-positive cells were counted using CellProfiler. HDV infection efficiency is shown either by infectious unit per mL (IU/mL) or HDV infection percentage $N = 5$ biological replicates from two independent experiments. (**D**) HLCs were transduced with or without AAV6-YFP or AAV6-NTCP. Two days post transduction, cell lysates were harvested and analyzed by Western blot analysis. (**E**) HLCs were infected with the indicated HDV genotype and 5 days p.i., cells were harvested for HDAg staining. Scale bar = 100 µm. (**F**) HLCs were infected with HDV (MOI = 5 Int. Units/cell) and harvested on indicated days p.i. HDV replication was analyzed by quantifying HDV genome copies in infected HLC lysates using RT-qPCR. Dashed line = LOQ. $N = 4$ biological replicates. (**G**) Huh7^{NTCP} cells were infected with HDV (MOI = 5 Units/cell). RNA lysates were harvested on day 1, 3, 5, 7, 9 p.i. HDV copy numbers of genomes and antigenomes were determined by strand-specific qRT-PCR, respectively. $N = 7$ biological replicates from two independent experiments. (**H**) HLCs, Huh7^{NTCP}, and differentiated HepaRG cells were uninfected or infected with HDV (MOI = 1 5 Units/cell).) or treated with BLV (500 nM). Cellular protein lysates were collected on day 1, 3, 5, 10, 15 p.i. and levels of L- and S-HDAg were analyzed by Western blot. Data information: In (**B**, **C**, **F** and **G**) data are presented as mean ± SD. Statistical significance in (**B**) was tested among HDV-infected HLC at different MOIs ($P < 0.0001$) by ordinary one-way ANOVA. Statistical significance in (**C**) was tested between HDV-infected HLC and dHepaRG ($P_{IU/mL} = 0.1384$; $P_{\%} = 0.0875$) and between HDV-infected HLC and Huh7^{NTCP} ($P_{IU/mL} < 0.0001$; $P_{\%} < 0.0001$) by multiple comparisons of ordinary one-way ANOVA. Statistical significance in (F) was tested between day 10- and day 15- harvested HDV-infected HLC ($P < 0.0001$) by multiple comparisons of ordinary one-way ANOVA. Statistical significance in (**G**) was tested between day 1- and day 3-harvested HDV-infected Huh7^{NTCP} ($P_{gRNA} < 0.0001$; $P_{agRNA} < 0.0001$) by an unpaired two-tailed *t* test. ****$P < 0.0001$, n.s.: non-significant.

© The Author(s)

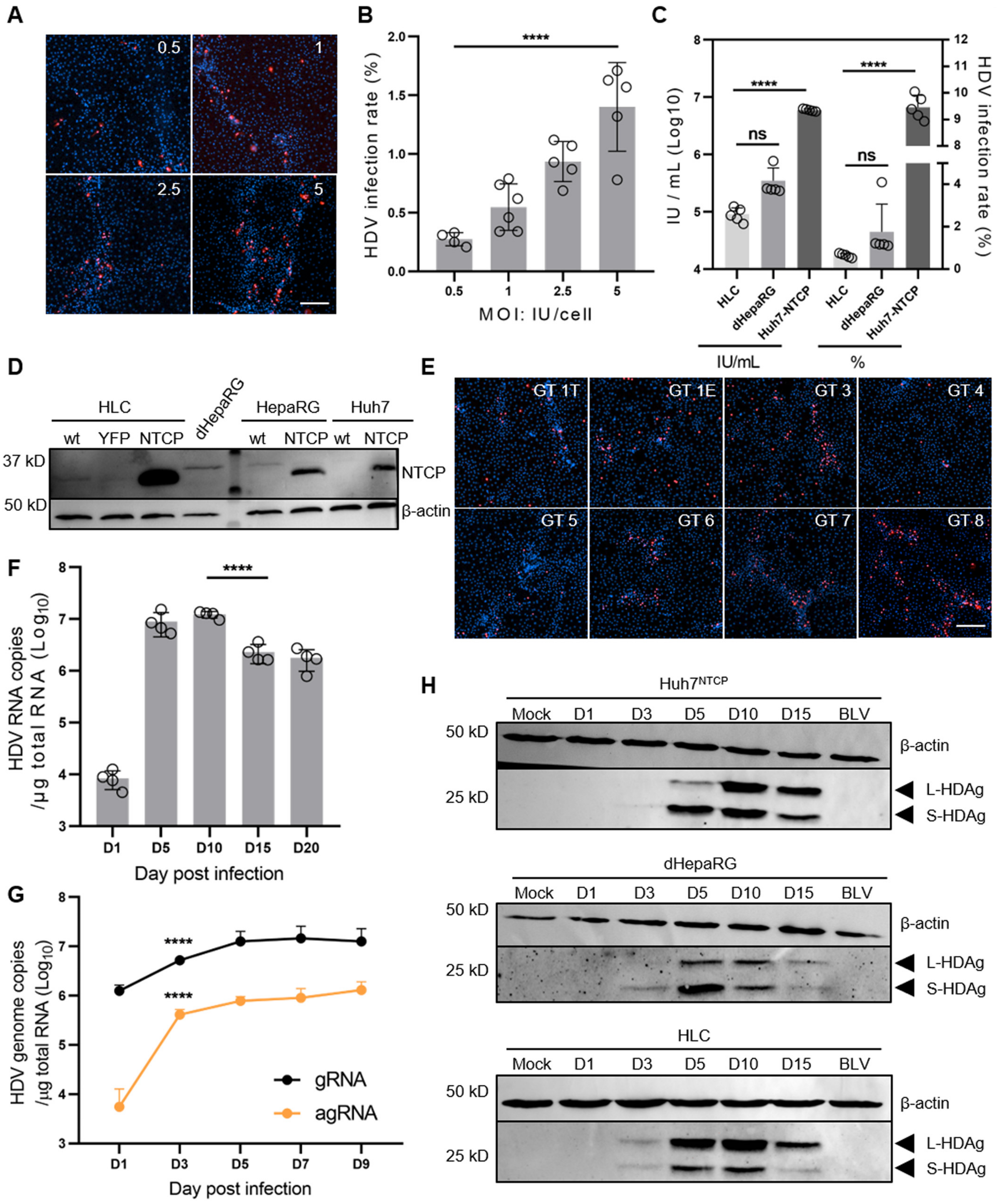
A
0.5
1
2.5
5
B
HDV infection rate (%)
MOI: IU/cell
C
IU / mL (Log10)
HDV infection rate (%)
HLC
dHepaRG
Huh7-NTCP
IU/mL
%
ns
D
HLC
dHepaRG
HepaRG
Huh7
wt
YFP
NTCP
37 kD
50 kD
β-actin
E
GT 1T
GT 1E
GT 3
GT 4
GT 5
GT 6
GT 7
GT 8
F
HDV RNA copies /μg total RNA (Log10)
Day post infection
G
HDV genome copies /μg total RNA (Log10)
gRNA
agRNA
Day post infection
H
Huh7NTCP
Mock
D1
D3
D5
D10
D15
BLV
25 kD
L-HDAg
S-HDAg
HLC

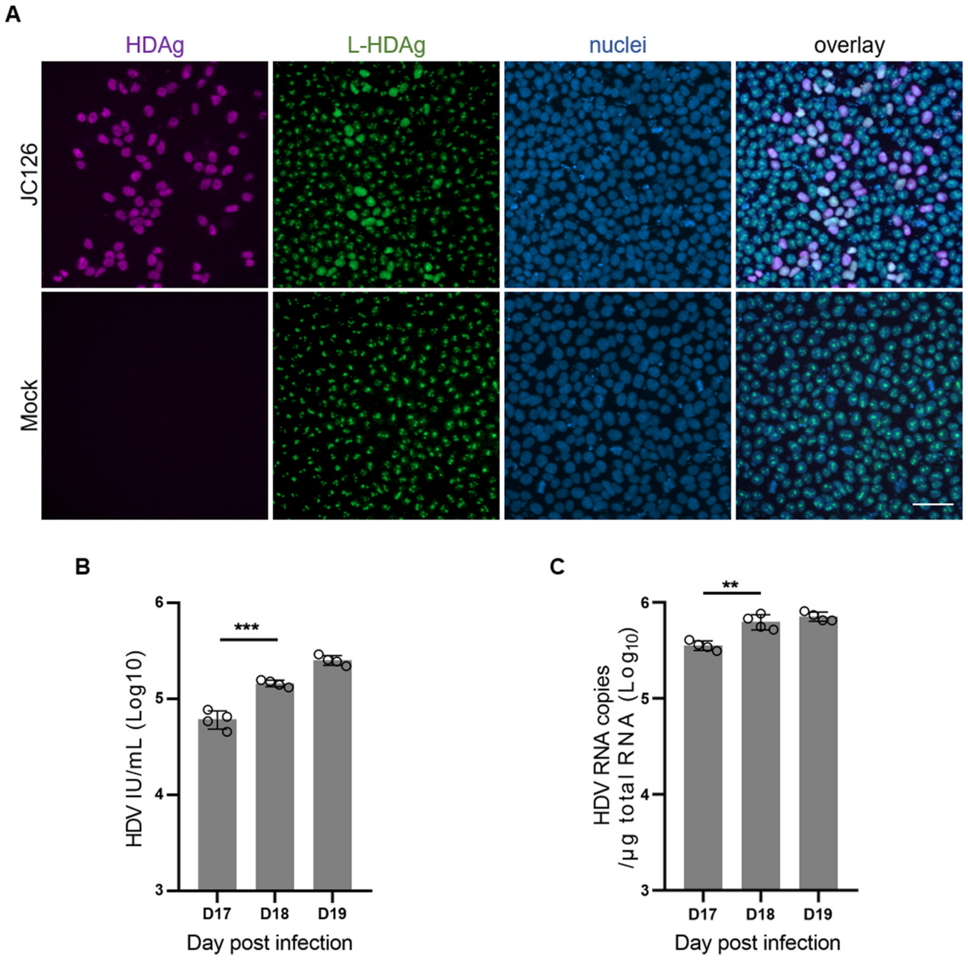

Figure EV2. HDV susceptibility along D17 to D19 of the HLC differentiation protocol.

(**A**) Undifferentiated WA09 cells were transfected with or without pJC126. Cells were harvested and assessed by immunofluorescence staining against the HDAg (magenta) and L-HDAg (green) 8 days post transfection. Scale bar = 50 µm. (**B**, **C**) HLCs were infected with HDV (MOI = 5) at the indicated day of the differentiation protocol and harvested for analyzing HDV infection efficiency by quantifying HDV-positive cells through CellProfiler (**B**) or detecting HDV total RNA copies through RT-qPCR (**C**) on 5 days p.i. $N = 4$ biological replicates from two independent HLC differentiations. Data information: In (**B**, **C**) data are presented as mean ± SD and statistical analysis was performed by multiple comparisons of ordinary one-way ANOVA. Statistical significance in (**B**) was tested between day 17- and day 18-harvested HDV-infected HLC ($P = 0.0004$). Statistical significance in (**C**) was tested between day 17- and day 18-harvested HDV-infected HLC ($P = 0.002$). ***$P < 0.001$; **$P < 0.01$.

 © The Author(s)

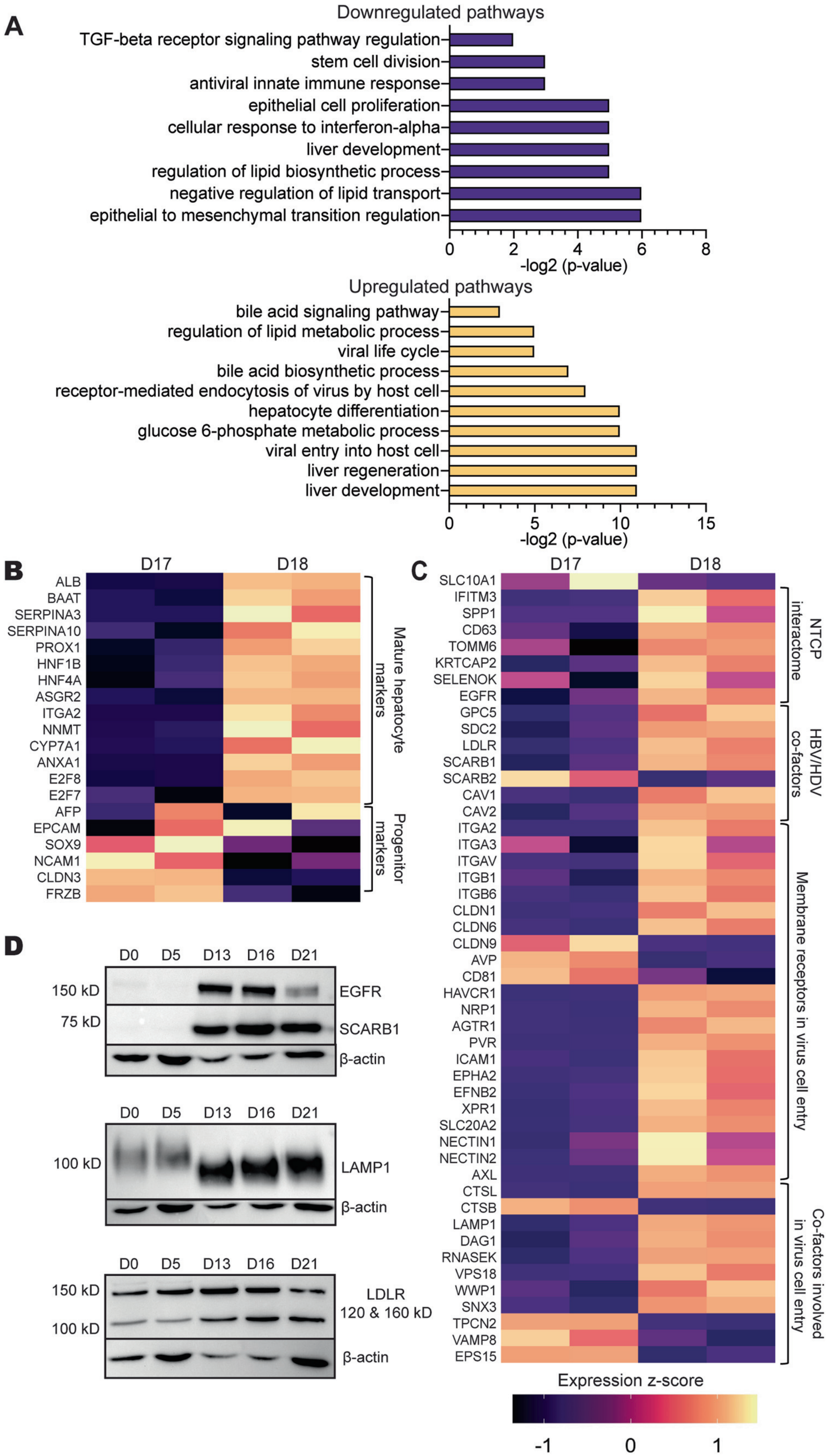
A
Downregulated pathways
TGF-beta receptor signaling pathway regulation
stem cell division
antiviral innate immune response
epithelial cell proliferation
cellular response to interferon-alpha
liver development
regulation of lipid biosynthetic process
negative regulation of lipid transport
epithelial to mesenchymal transition regulation
0 2 4 6 8
-log2 (p-value)
Upregulated pathways
bile acid signaling pathway
regulation of lipid metabolic process
viral life cycle
bile acid biosynthetic process
receptor-mediated endocytosis of virus by host cell
hepatocyte differentiation
glucose 6-phosphate metabolic process
viral entry into host cell
liver regeneration
liver development
0 5 10 15
-log2 (p-value)
B
D17 D18
ALB BAAT SERPINA3 SERPINA10 PROX1 HNF1B HNF4A ASGR2 ITGA2 NNMT CYP7A1 ANXA1 E2F8 E2F7
Mature hepatocyte markers
AFP EPCAM SOX9 NCAM1 CLDN3 FRZB
Progenitor markers
C
D17 D18
SLC10A1 IFITM3 SPP1 CD63 TOMM6 KRTCAP2 SELENOK EGFR
NTCP interactome
GPC5 SDC2 LDLR SCARB1 SCARB2 CAV1 CAV2
HBV/HDV co-factors
ITGA2 ITGA3 ITGAV ITGB1 ITGB6 CLDN1 CLDN6 CLDN9 AVP CD81 HAVCR1 NRP1 AGTR1 PVR ICAM1 EPHA2 EFNB2 XPR1 SLC20A2 NECTIN1 NECTIN2 AXL
Membrane receptors in virus cell entry
CTSL CTSB LAMP1 DAG1 RNASEK VPS18 WWP1 SNX3 TPCN2 VAMP8 EPS15
Co-factors involved in virus cell entry
Expression z-score
-1 0 1
D
D0 D5 D13 D16 D21
150 kD EGFR
75 kD SCARB1
β-actin
D0 D5 D13 D16 D21
100 kD LAMP1
β-actin
D0 D5 D13 D16 D21
150 kD
100 kD
LDLR
120 & 160 kD
β-actin

Figure EV3. Gene expression along HLC differentiation.

Total RNA was extracted from HLCs at either day 17 or 18 during the differentiation protocol and subjected to whole-transcriptome expression profiling and gene ontology (GO) term enrichment analysis. (**A**) GO term enrichment analysis of biological pathways for up- and downregulated genes between HLCs at day 17 and 18. Differentially expressed genes (P value < 0.05) were significantly enriched in this GO term. Statistical analysis was performed using the Kolmogorov–Smirnov test. (**B**–**D**) Heatmap of Z score-normalized counts per million (CPM) values for (**B**) hepatocyte markers, and (**C**) virus entry factors. $N = 2$ biological replicates. (**D**) Cellular protein lysates of mature HLCs were collected and expression levels of previously described HBV/HDV entry factors and cofactors were analyzed by western blot.

 © The Author(s)

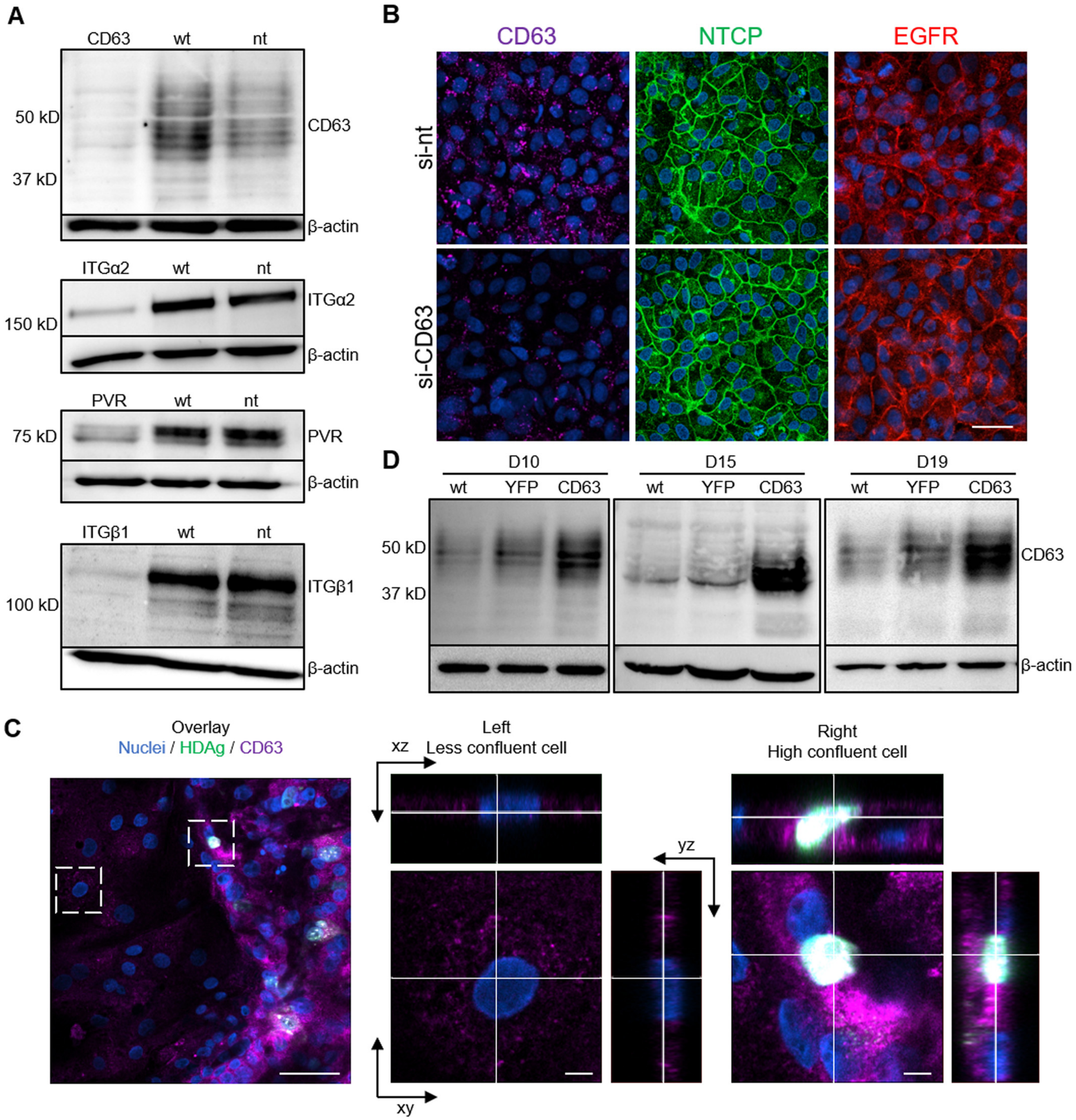

Figure EV4. Knockdown of CD63 does not alter membranous NTCP expression.

(**A**) Four siRNAs targeting CD63, ITGA2, PVR, and ITGB1 were delivered into Huh7[NTCP] cells and downregulation was confirmed by Western blot analysis on cell lysates harvested 3 days post transfection. Untransfected (wt) and non-target (nt) siRNAs were used as controls. (**B**) Huh7[NTCP] cells were transfected with 50 nM siRNA targeting CD63. 3 days later, NTCP protein stained with Atto-MyrB-488 (green), CD63 and EGFR stained with specific antibody were imaged by confocal microscopy. Scale bar = 50 µm. (**C**) Left: Mature HLCs were infected with HDV, fixed 5 days p.i., and stained for CD63 (magenta), HDAg (green), and the nucleus (blue). Images were taken on the Airyscan confocal microscope. Left: Shown is a 40x tile image with the two HLC populations (reused from Fig. 5G, merge). Scale bar = 50 µm. Middle & Right: Zoom in image of the region of interest. Single z-slice and orthogonal xz and yz views of less (middle) and high (right) confluent HLCs. Scale bar = 5 µm. (**D**) Endogenous and ectopic CD63 expression at the different hepatocyte differentiation stages. Cells were transduced with AAV6s two days before harvesting them at indicated day of the differentiation protocol. Lysates were analyzed by Western blot using specific anti-CD63 and β-actin antibodies.

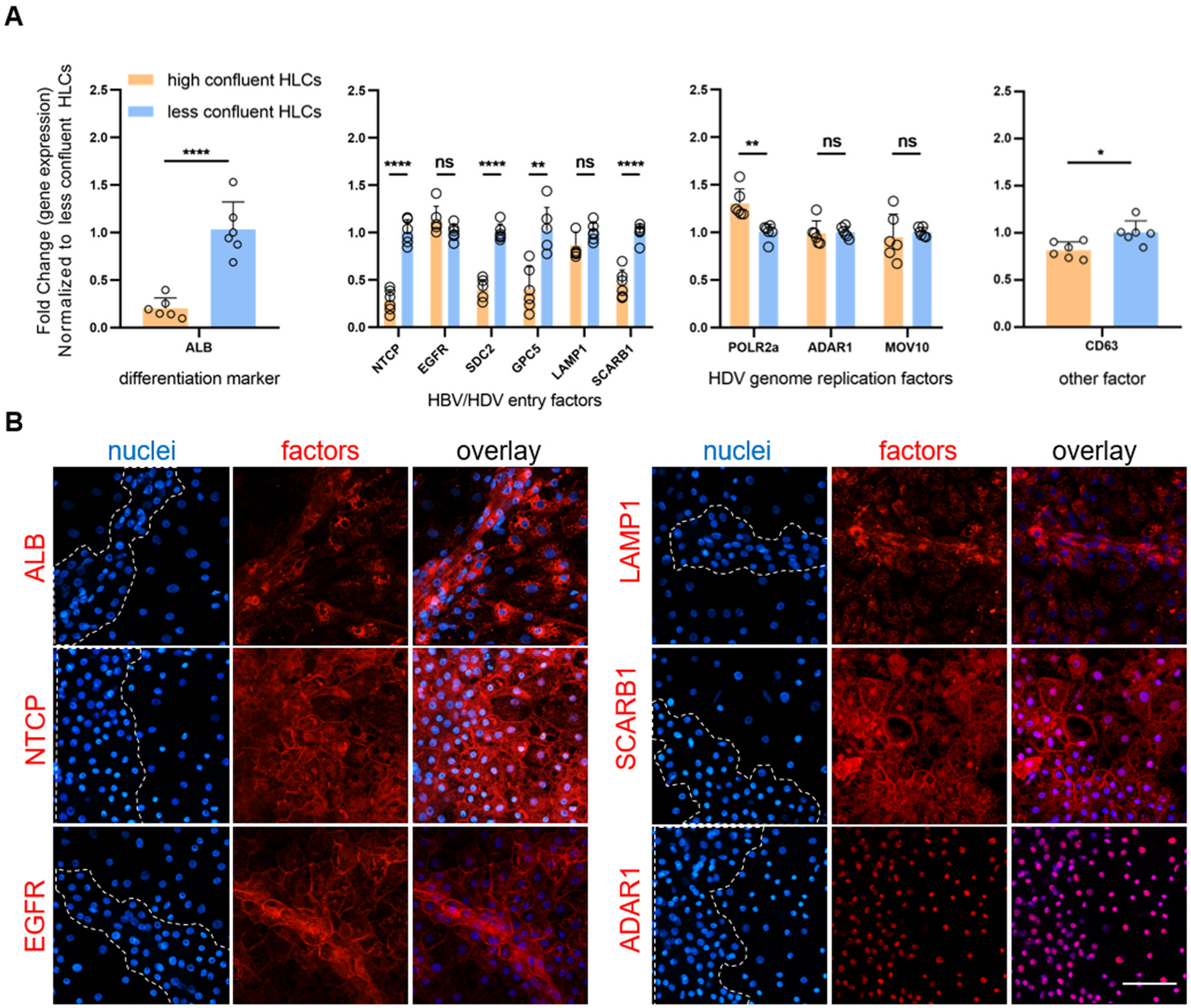

Figure EV5. Transcriptional and protein levels of host factors in high and less confluent HLC populations.

(**A**) Cellular lysates were harvested from high and less confluent HLC populations. Differentiation factor *ALB*, entry factors (*NTCP, EGFR, SDC2, GPC5, LAMP1, SCARB1*), HDV genome replication factor (*POLR2A, ADAR1, MOV10)* and other virus–host interaction factors (CD63) were analyzed using RT-qPCR. *N* = 6 biological replicates from two independent HLC differentiations. (**B**) Selected factors in (**A**) were immunostained in HLCs (shown in red). Images were taken on the Airyscan confocal microscope. Shown are 40× tile image with the two HLC populations. Scale bar = 50 µm. Highly confluent HLCs are indicated by the white dashed line. Data information: In (**A**) data are presented as mean ± SD and statistical analysis was performed by unpaired two-tailed *t* test. Statistical significance was tested between high and less confluent HLC ($P_{ALB} < 0.0001$; $P_{NTCP} < 0.0001$; $P_{EGFR} = 0.0950$; $P_{SDC2} < 0.0001$; $P_{GPC5} = 0.0012$; $P_{LAMP1} = 0.0738$; $P_{SCARB1} < 0.0001$; $P_{POLR2a} = 0.0018$; $P_{ADAR1} = 0.08617$; $P_{MOV10} = 0.6186$; $P_{ALB} = 0.0117$). ****$P < 0.0001$; **$P < 0.01$; *$P < 0.05$; n.s.: non-significant.

 © The Author(s)
